# Supplementary material for: Integrin β1 coordinates survival and morphogenesis of the embryonic lineage upon implantation and pluripotency transition
Source: Cell Rep. 2021 Mar 9;34(10):108834. doi: 10.1016/j.celrep.2021.108834 (PMC7966855; doi:10.1016/j.celrep.2021.108834)
Supplement: Document S1. Figures S1–S3 [file mmc1.pdf]

Cell Reports, Volume *34*

Supplemental information

Integrin β1 coordinates survival and morphogenesis of the embryonic lineage upon implantation and pluripotency transition

Matteo Amitaba Molè, Antonia Weberling, Reinhard Fässler, Alison Campbell, Simon Fishel, and Magdalena Zernicka-Goetz

SUPPLEMENTAL INFORMATION:

Integrin β1 coordinates survival and morphogenesis of the embryonic lineage upon implantation and pluripotency transition

Matteo Amitaba Molè[1†], Antonia Weberling[1†], Reinhard Fässler[2], Alison Campbell[3], Simon Fishel[3,4], Magdalena Zernicka-Goetz[1,5,6,*]

Figure S1

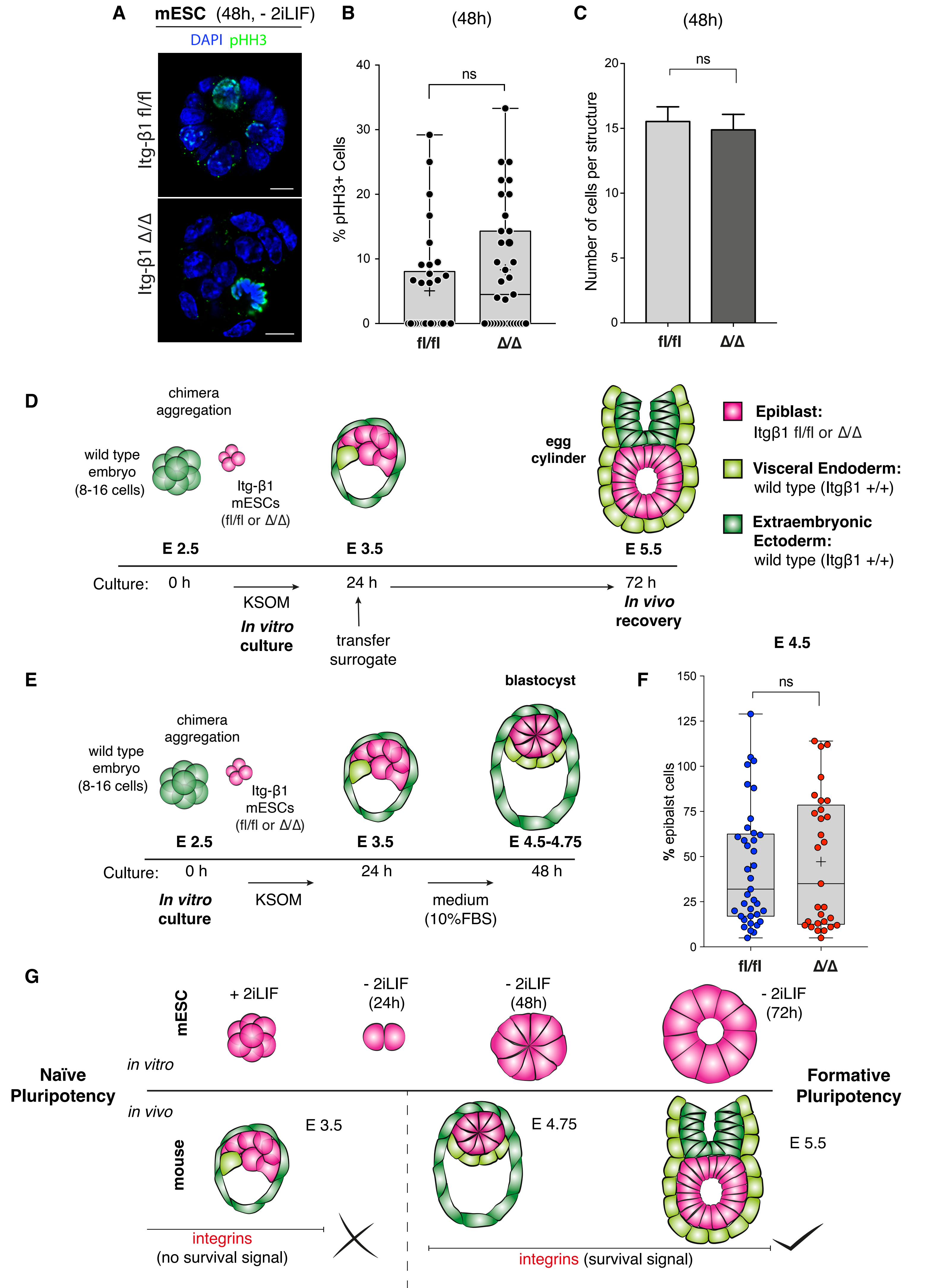
A
mESC (48h, - 2iLIF)
DAPI pHH3
Itg-β1 fl/fl
Itg-β1 Δ/Δ
B
(48h)
ns
% pHH3+ Cells
0
10
20
30
40
fl/fl
Δ/Δ
C
(48h)
ns
Number of cells per structure
0
5
10
15
20
fl/fl
Δ/Δ
D
chimera aggregation
wild type embryo (8-16 cells)
Itg-β1 mESCs (fl/fl or Δ/Δ)
egg cylinder
E 2.5
E 3.5
E 5.5
Culture:
0 h
KSOM
In vitro culture
24 h
transfer surrogate
72 h
In vivo recovery
Epiblast: Itgβ1 fl/fl or Δ/Δ
Visceral Endoderm: wild type (Itgβ1 +/+)
Extraembryonic Ectoderm: wild type (Itgβ1 +/+)
E
chimera aggregation
wild type embryo (8-16 cells)
Itg-β1 mESCs (fl/fl or Δ/Δ)
blastocyst
E 2.5
E 3.5
E 4.5-4.75
Culture:
0 h
KSOM
In vitro culture
24 h
medium (10%FBS)
48 h
F
E 4.5
ns
% epibalst cells
0
25
50
75
100
125
150
fl/fl
Δ/Δ
G
mESC
+ 2iLIF
- 2iLIF (24h)
- 2iLIF (48h)
- 2iLIF (72h)
in vitro
Naïve Pluripotency
Formative Pluripotency
in vivo
mouse
E 3.5
E 4.75
E 5.5
integrins (no survival signal)
integrins (survival signal)

Figure S1. Proliferation and methods for the generation of chimeric embryos. Related to Figure 1. (**A**), Cells undergoing mitosis as revealed by phospho-histone H3 staining in wild type (fl/fl, upper) and mutant (Δ/Δ, lower) mESC spheroids at 48-hours culture. Scale bars: 10 μm. (**B**), Quantification of mitotic index as the percentage of phospho-histone H3 positive cells of the total cell number in each spheroid. Mann-Whitney Test, p=ns, number of spheroids n=34 (fl/fl), n=35 (Δ/Δ); number of replicates=3. (**C**), Comparison of the total number of cells in each spheroid. Mann-Whitney Test, p=ns. (**D**), Embryos recovered at E2.5 (8-16 cells stage) from wild type embryos are aggregated with mESCs either wild type (fl/fl) or mutant (Δ/Δ) for *Itgβ1* locus. Embryos are cultured for 24-hours in KSOM and then transferred into E2.5 pseudo-pregnant surrogate mice. After 72-hours embryos are recovered by dissection. Extraembryonic lineages (ExE and VE) are derived from wild type embryos, while the epiblast compartment from mESCs homozygous for either the floxed (fl/fl) or deleted allele (Δ/Δ) of *Itgβ1*. (**E**), Culture method to generate chimeric embryos at the expanded blastocyst stage E4.5-4.75: embryos recovered at E2.5 (8-16 cells stage) from wild type embryos are aggregated with mESC either wild type (fl/fl) or mutant (Δ/Δ), cultured for 24-hours in KSOM and transferred into IVC (*in vitro* culture) medium supplemented with 10% FBS for further 24-hours. (**F**), Comparison of the total number of epiblast cells in chimeric blastocysts at E4.5-4.75 between wild types (Epi:fl/fl) and mutants (Epi:Δ/Δ) as refereed Figure 1 H-J: Mann-Whitney Test, p=ns (number of embryos n=37 (fl/fl), n=29 (Δ/Δ)). (**G**), Schematics of integrin requirement throughout development in mESCs (upper) and mouse embryos (lower). Integrin signalling is dispensable during early pre-implantation development corresponding to the *in vitro* state of naïve pluripotency. Conversely, signaling from integrin-mediated adhesion ensures survival of the epiblast upon implantation and transition towards the formative state of pluripotency. Scale bar: 10 μm (A).

Figure S2

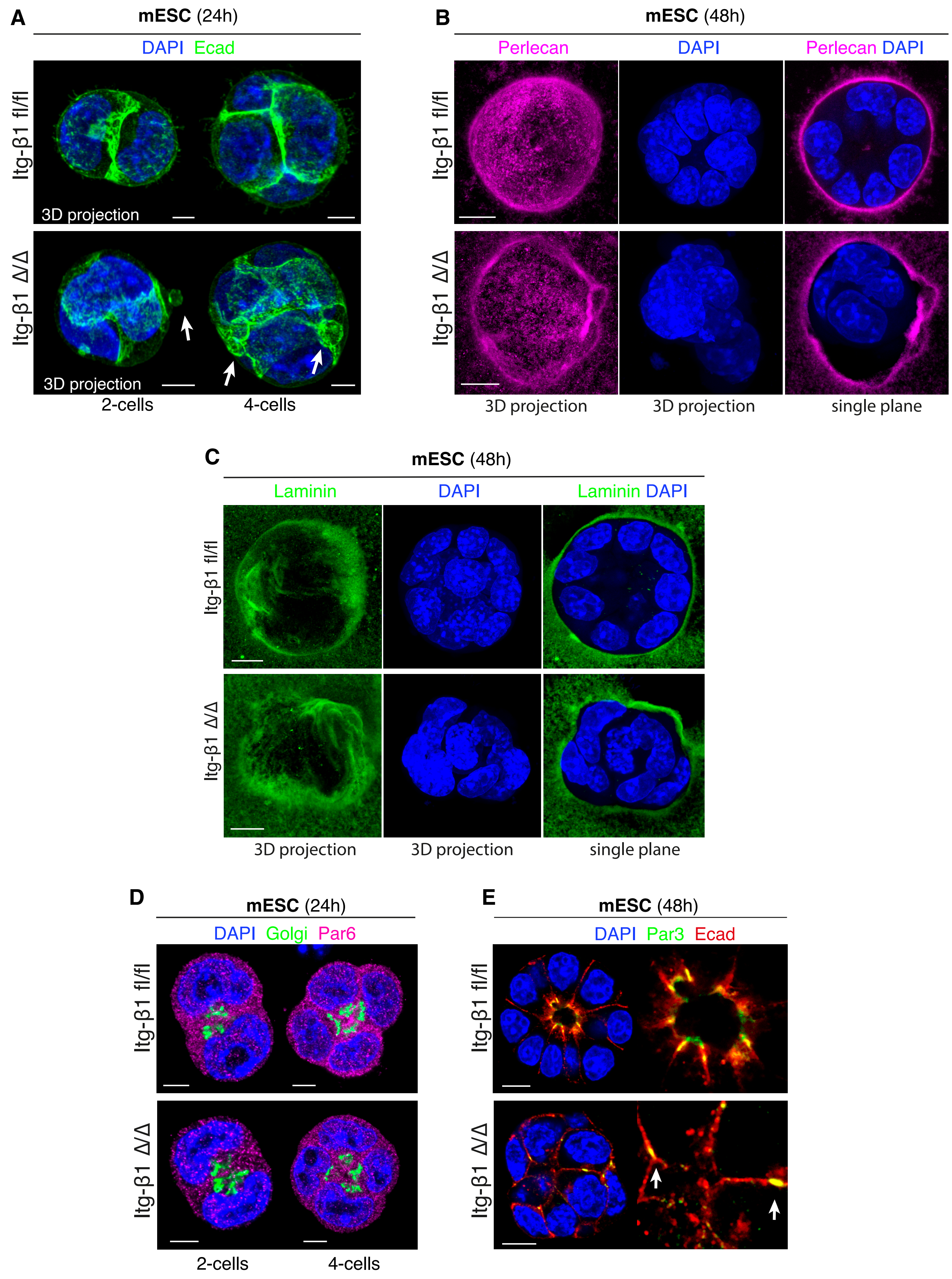
A
mESC (24h)
DAPI Ecad
Itg-β1 fl/fl
Itg-β1 Δ/Δ
3D projection
3D projection
2-cells
4-cells
B
mESC (48h)
Perlecan
DAPI
Perlecan DAPI
Itg-β1 fl/fl
Itg-β1 Δ/Δ
3D projection
3D projection
single plane
C
mESC (48h)
Laminin
DAPI
Laminin DAPI
Itg-β1 fl/fl
Itg-β1 Δ/Δ
3D projection
3D projection
single plane
D
mESC (24h)
DAPI Golgi Par6
Itg-β1 fl/fl
Itg-β1 Δ/Δ
2-cells
4-cells
E
mESC (48h)
DAPI Par3 Ecad
Itg-β1 fl/fl
Itg-β1 Δ/Δ

Figure S2. Loss of integrin β1 causes basal blebs but is dispensable for apico-basal polarity establishment or basement membrane assembly. Related to Figure 3. (**A**), Blebs are observed after 24-hours culture on the basal domain of mutant mESCs (arrows). (**B**-**C**), Immunofluorescences staining for the basement membrane (BM) components Perlecan (B) and pan-Laminins (C) at 48-hours culture. Mutant mESCs (Itgβ1 Δ/Δ) assemble correctly the BM on the basal side, similarly to wild types (Itgβ1 fl/fl). (**D**), Both wild type and mutant cells orient the Golgi apparatus towards the apical site. PAR6 complex is not expressed at 24-hours. (**E**) Assessment of polarity at 48-hours by the localization of PAR3. In wild type cells, PAR3 is recruited at apical junctions at the site of E-cadherin expression. In mutant cells, PAR3 maintains a junctional localization, as opposed to basal PAR6 (see Figure 4H). Scale bars: 5 μm (A, D), 10 μm (B-C, E).

Figure S3

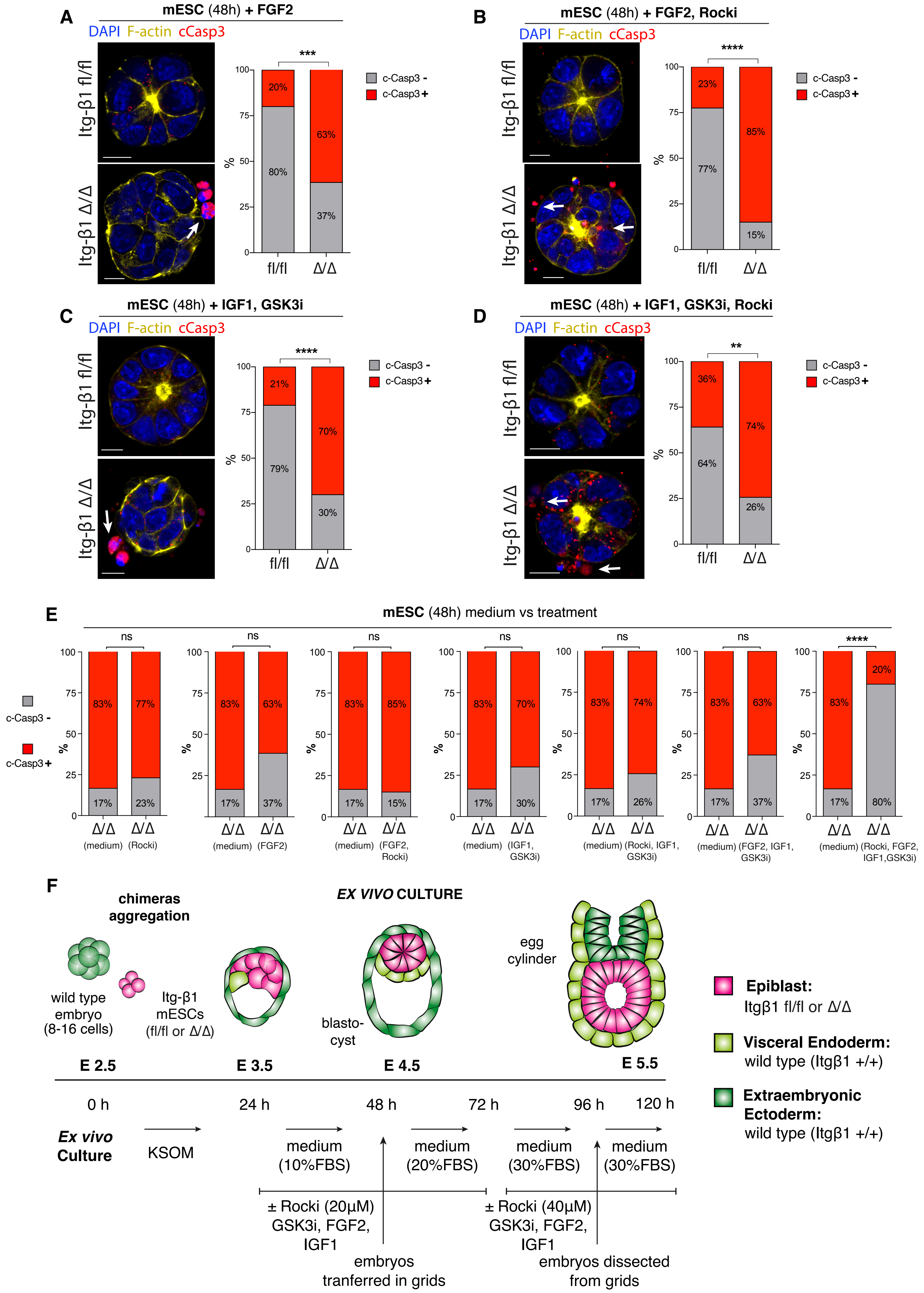
A
mESC (48h) + FGF2
DAPI F-actin cCasp3
Itg-β1 fl/fl
Itg-β1 Δ/Δ
***
20%
80%
63%
37%
%
fl/fl
Δ/Δ
c-Casp3 -
c-Casp3 +
B
mESC (48h) + FGF2, Rocki
DAPI F-actin cCasp3
Itg-β1 fl/fl
Itg-β1 Δ/Δ
****
23%
77%
85%
15%
%
fl/fl
Δ/Δ
c-Casp3 -
c-Casp3 +
C
mESC (48h) + IGF1, GSK3i
DAPI F-actin cCasp3
Itg-β1 fl/fl
Itg-β1 Δ/Δ
****
21%
79%
70%
30%
%
fl/fl
Δ/Δ
c-Casp3 -
c-Casp3 +
D
mESC (48h) + IGF1, GSK3i, Rocki
DAPI F-actin cCasp3
Itg-β1 fl/fl
Itg-β1 Δ/Δ
**
36%
64%
74%
26%
%
fl/fl
Δ/Δ
c-Casp3 -
c-Casp3 +
E
mESC (48h) medium vs treatment
c-Casp3 -
c-Casp3 +
ns
83% 77%
17% 23%
Δ/Δ (medium) Δ/Δ (Rocki)
ns
83% 63%
17% 37%
Δ/Δ (medium) Δ/Δ (FGF2)
ns
83% 85%
17% 15%
Δ/Δ (medium) Δ/Δ (FGF2, Rocki)
ns
83% 70%
17% 30%
Δ/Δ (medium) Δ/Δ (IGF1, GSK3i)
ns
83% 74%
17% 26%
Δ/Δ (medium) Δ/Δ (Rocki, IGF1, GSK3i)
ns
83% 63%
17% 37%
Δ/Δ (medium) Δ/Δ (FGF2, IGF1, GSK3i)
****
83% 20%
17% 80%
Δ/Δ (medium) Δ/Δ (Rocki, FGF2, IGF1,GSK3i)
F
chimeras aggregation
EX VIVO CULTURE
wild type embryo (8-16 cells)
Itg-β1 mESCs (fl/fl or Δ/Δ)
blasto-cyst
egg cylinder
E 2.5
E 3.5
E 4.5
E 5.5
0 h
24 h
48 h
72 h
96 h
120 h
Ex vivo Culture
KSOM
medium (10%FBS)
medium (20%FBS)
medium (30%FBS)
medium (30%FBS)
± Rocki (20µM) GSK3i, FGF2, IGF1
± Rocki (40µM) GSK3i, FGF2, IGF1
embryos tranferred in grids
embryos dissected from grids
Epiblast: Itgβ1 fl/fl or Δ/Δ
Visceral Endoderm: wild type (Itgβ1 +/+)
Extraembryonic Ectoderm: wild type (Itgβ1 +/+)

Figure S3. Pharmacological treatments, impact on morphogenesis and survival. Related to Figure 5 and 6. (**A**), Supplementation of FGF2 alone does not prevent initiation of apoptosis in integrin β1 mutant cells (Δ/Δ): Fisher's Exact Test: *** p=0.0004 (number of spheroids n=35 (fl/fl), n=39 (Δ/Δ), 3 replicate experiments). (**B**), Supplementation of FGF2 + Rock inhibitor does not prevent initiation of apoptosis in integrin β1 mutant cells (Δ/Δ): Fisher's Exact Test: **** p<0.0001 (number of spheroids n=40 (fl/fl), n=40 (Δ/Δ), 3 replicate experiments). (**C**), Supplementation of IGF1 + GSK3 inhibitor does not prevent initiation of apoptosis in integrin β1 mutant cells (Δ/Δ): Fisher's Exact Test: **** p<0.0001 (number of spheroids n=38 (fl/fl), n=40 (Δ/Δ), 3 replicate experiments). (**D**), Supplementation of IGF1 + GSK3 inhibitor + Rock inhibitor does not prevent initiation of apoptosis in integrin β1 mutant cells (Δ/Δ): Fisher's Exact Test: ** p=0.0013 (number of spheroids n=39 (fl/fl), n=39 (Δ/Δ), 3 replicate experiments). (**E**) Comparison of the Δ/Δ mESCs in medium without supplementation vs. all conditions tested at 48-hours: from left to right Rocki alone, FGF2 alone, FGF2+Rocki, IGF1+GSK3i, Rocki+IGF1+GSK3i, FGF2+ IGF1+GSK3i, and complete supplementation with Rocki+FGF2+ IGF1+GSK3i. Treatment with each combination is not able to rescue the survival and does not differ from Δ/Δ mESCs in medium only (Fisher's Exact Test: p=ns). Only the supplementation of the four factors together (Rocki+FGF2+ IGF1+GSK3i) is able to rescue the survival of the mutant mESCs (Δ/Δ in medium vs Δ/Δ in full supplementation: Fisher's Exact Test: ****p<0.0001), to a comparable level to wild type (Figure 5D, Fisher's Exact Test: p=ns). (**F**), Method of culture from pre- to post implantation: blastocysts are recovered at E2.5 (8-16 cells stage) from wild type embryos and aggregated with mESC wild type (fl/fl) or mutant (Δ/Δ) for *Itgβ1* locus. Embryos are cultured for 24-hours in KSOM in drops. Expanded blastocysts are moved in grid mesh in medium with increasing concentration of FBS supplemented as shown. FGF2, IGF1 and GSK3i are supplemented in medium after 24-hours. ROCKi is supplemented in medium after 24-hours at 20 μM and increased to 40 μm during the last 48-hours culture. See methods for more details. Scale bars: 10 μm (A-E)
