# Supplementary material for: Integrin β1 coordinates survival and morphogenesis of the embryonic lineage upon implantation and pluripotency transition
Source: Cell Rep. 2021 Mar 9;34(10):108834. doi: 10.1016/j.celrep.2021.108834 (PMC7966855; doi:10.1016/j.celrep.2021.108834)
Supplement: Document S2. Article plus supplemental information [file mmc4.pdf]

Cell Reports

Article

Integrin β1 coordinates survival and morphogenesis of the embryonic lineage upon implantation and pluripotency transition

Graphical Abstract

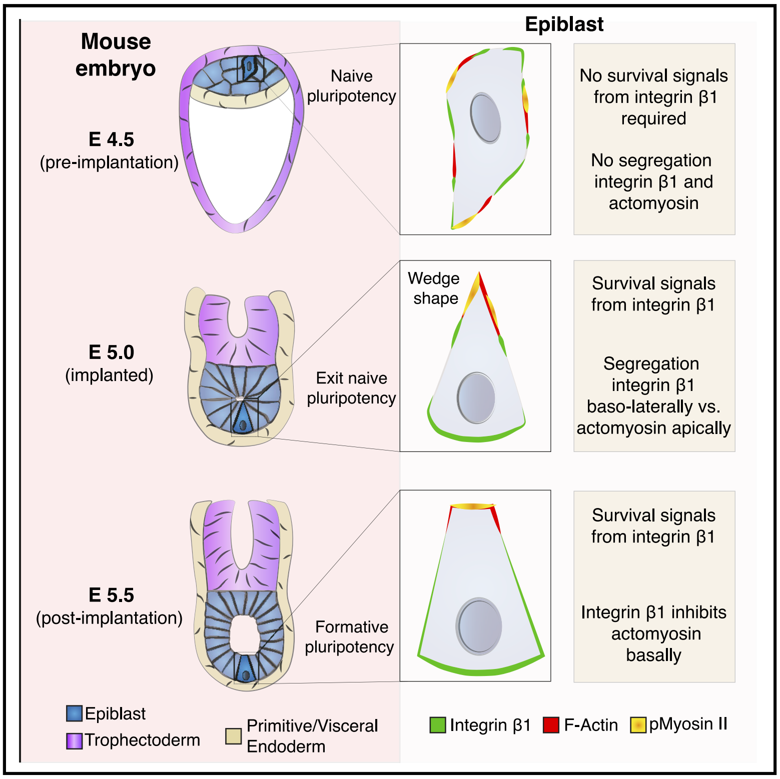

Highlights

- Integrin β1 is required for epiblast survival upon implantation
- Integrin β1 regulates epiblast morphogenesis by basal actomyosin inhibition
- Prosurvival signals and local actomyosin suppression enables epiblast development

Authors

Matteo Amitaba Molè, Antonia Weberling, Reinhard Fässler, Alison Campbell, Simon Fishel, Magdalena Zernicka-Goetz

Correspondence

mz205@cam.ac.uk

In brief

Molè et al. show that the coordinated stimulation of pro-survival signals together with ROCK-mediated localized suppression of actomyosin downstream of integrin β1 regulate morphogenesis and survival of the embryonic lineage during the transition from pre- to post-implantation.

Molè et al., 2021, Cell Reports 34, 108834
March 9, 2021 © 2021 The Authors.
https://doi.org/10.1016/j.celrep.2021.108834

CellPress

Cell Reports

CellPress OPEN ACCESS

Article

Integrin β1 coordinates survival and morphogenesis of the embryonic lineage upon implantation and pluripotency transition

Matteo Amitaba Molè,[1,6] Antonia Weberling,[1,6] Reinhard Fässler,[2] Alison Campbell,[3] Simon Fishel,[3,4] and Magdalena Zernicka-Goetz[1,5,7,*]

[1]Mammalian Embryo and Stem Cell Group, Department of Physiology, Development, and Neuroscience, University of Cambridge, Downing Street, Cambridge CB2 3EG, UK

[2]Department of Molecular Medicine, Max Planck Institute of Biochemistry, Am Klopferspitz 18, 82152 Martinsried, Germany

[3]CARE Fertility Group, John Webster House, 6 Lawrence Drive, Nottingham Business Park, Nottingham NG8 6PZ, UK

[4]School of Pharmacy and Biomolecular Sciences, Liverpool John Moores University, Byrom Street, Liverpool L3 3AF, UK

[5]Plasticity and Self-Organization Group, Division of Biology and Biological Engineering, California Institute of Technology (Caltech), Pasadena, CA 91125, USA

[6]These authors contributed equally

[7]Lead contact

*Correspondence: mz205@cam.ac.uk

https://doi.org/10.1016/j.celrep.2021.108834

SUMMARY

At implantation, the embryo establishes contacts with the maternal endometrium. This stage is associated with a high incidence of preclinical pregnancy losses. While the maternal factors underlying uterine receptivity have been investigated, the signals required by the embryo for successful peri-implantation development remain elusive. To explore these, we studied integrin β1 signaling, as embryos deficient for this receptor degenerate at implantation. We demonstrate that the coordinated action of pro-survival signals and localized actomyosin suppression via integrin β1 permits the development of the embryo beyond implantation. Failure of either process leads to developmental arrest and apoptosis. Pharmacological stimulation through fibroblast growth factor 2 (FGF2) and insulin-like growth factor 1 (IGF1), coupled with ROCK-mediated actomyosin inhibition, rescues the deficiency of integrin β1, promoting progression to post-implantation stages. Mutual exclusion between integrin β1 and actomyosin seems to be conserved in the human embryo, suggesting the possibility that these mechanisms could also underlie the transition of the human epiblast from pre- to post-implantation.

INTRODUCTION

Implantation is a critical stage of mammalian embryogenesis (Aplin and Ruane, 2017; Cha et al., 2012; Geisert and Fuller, 2015). Up to 30% of human embryos fail to complete implantation, resulting in spontaneous abortions (Koot et al., 2012; Macklon et al., 2002; Wilcox et al., 1988, 1999). Successful implantation is the result of a coordinated crosstalk between the developing blastocyst and the maturation of the endometrium (Lucas et al., 2016; Norwitz et al., 2001). While the molecular factors underlying uterine receptivity have been extensively investigated (Cakmak and Taylor, 2011; Cha et al., 2012; Koot et al., 2012), the signaling necessary for the embryo proper to develop through implantation remains largely unexplored. To gain insights into these molecular mechanisms, we decided to study the integrin β1 mutant mouse, as embryos genetically deficient for integrin β1 arrest at the time of implantation (Fässler and Meyer, 1995; Moore et al., 2014; Stephens et al., 1995).

Integrin β1 is a central member of a large family of heterodimeric cell surface receptors, which act as primary linkage between the extracellular environment and the internal cytoskeleton of cells to regulate numerous cellular responses (Barczyk et al., 2010; Campbell and Humphries, 2011; Kechagia et al., 2019; Schwartz, 2010). While embryos deficient for integrin β1 successfully implant into the endometrium, they fail to progress beyond this stage (Stephens et al., 1995). A similar phenotype of embryonic peri-implantation lethality could be observed when the integrin ligands laminin β1 (Miner et al., 2004) and laminin γ1 (Smyth et al., 1999) or key downstream factors such as integrin-linked kinase (ILK) (Sakai et al., 2003) and Pinch1 (Li et al., 2005) were lost, pointing toward a critical requirement for integrin signaling during progression of the mammalian embryo beyond implantation (Sutherland et al., 1993). Alongside lethality, loss of integrin-mediated signaling has been correlated with the inability of cells to organize into polarized structures, both in the context of embryonic stem cells (ESCs) as well as in models of *in vivo* and *in vitro* epithelial

This is an open access article under the CC BY license (http://creativecommons.org/licenses/by/4.0/).

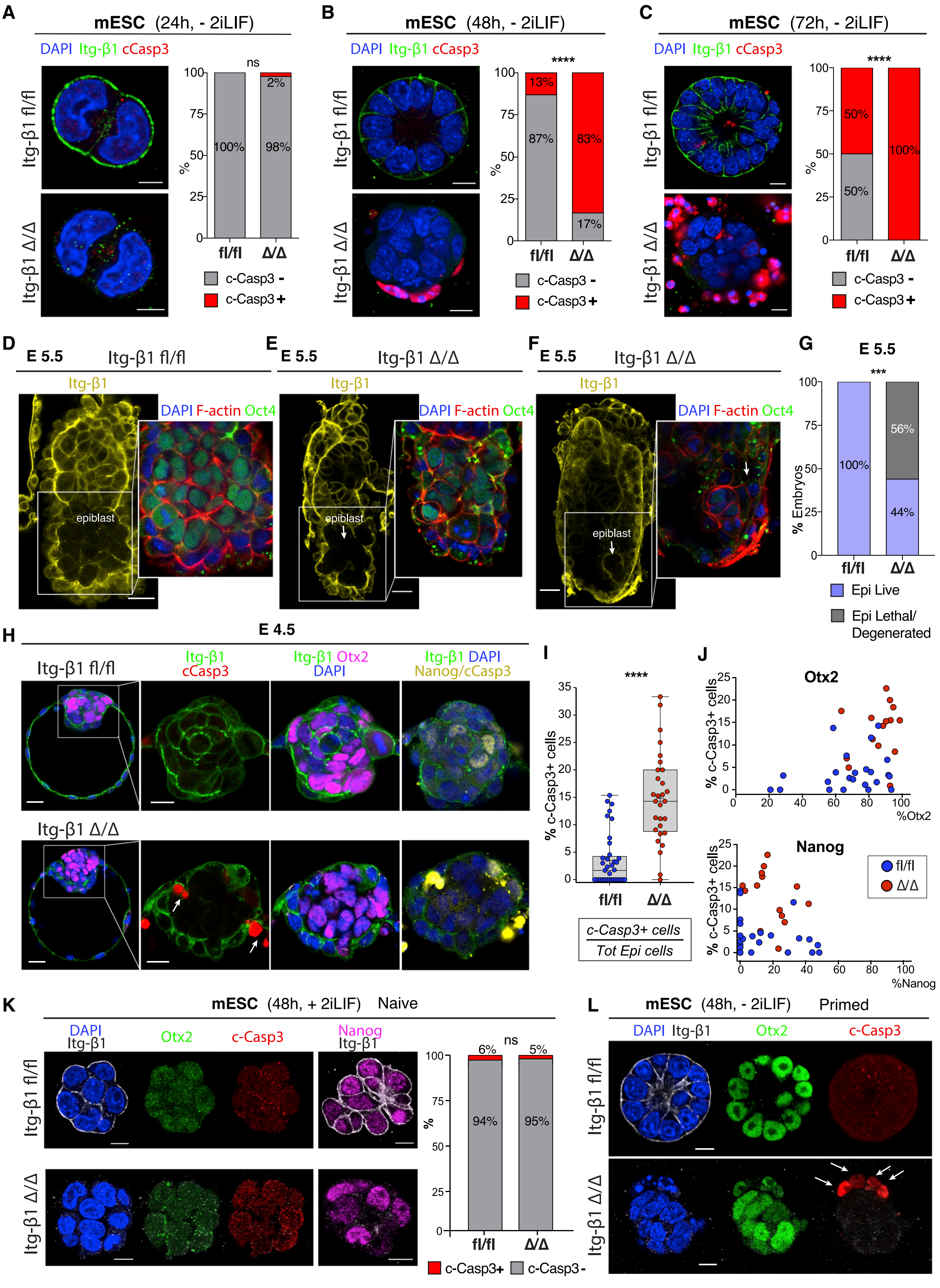

(legend on next page)

morphogenesis (Akhtar and Streuli, 2013; Aumailley et al., 2000; Li et al., 2017; Moore et al., 2014; Yu et al., 2005).

During the transition from pre- to post-implantation, the epiblast, the primary lineage for the formation of the embryo proper, transforms from a group of nonpolar, disorganized cells into a structured epithelium, with cells arranging around a shared point of apical constriction (Bedzhov and Zernicka-Goetz, 2014; Luckett, 1975; Wallingford et al., 2013). This rosette-like configuration progresses to then form the mature post-implantation epiblast, a monolayered polarized epithelium surrounding a cavity at its center (Molè et al., 2020). Alongside this substantial morphogenetic transformation, the epiblast undergoes transcriptional changes by dismantling the pluripotency genes of the naive state and acquiring a lineage-biased formative/primed state after implantation (Boroviak and Nichols, 2017; Hackett and Surani, 2014; Kinoshita et al., 2020; Wu et al., 2016).

Here, we use the integrin β1 conditional mutant to gain insights into the role of integrin signaling for the development of the mammalian embryo during the pre- to post-implantation transition. Our study indicates that integrin-mediated signaling coordinates cytoskeleton remodeling and survival to promote development of the epiblast during implantation and pluripotency transition.

RESULTS

To explore the molecular process leading to lethality of the embryo in the absence of integrin β1, we derived a conditional mouse embryonic stem cell (mESC) line from homozygous embryos carrying the *Itgβ1* floxed allele (Potocnik et al., 2000) and cultured them for 24, 48, and 72 h under differentiating conditions in 3D to recapitulate peri-implantation morphogenesis of the embryonic lineage *in vitro* (Shahbazi and Zernicka-Goetz, 2018). Wild-type cells carrying the unrecombined allele of *Itgβ1* (*fl/fl)* developed as previously shown and gave rise to a rosette-like structure surrounding a central cavity (Bedzhov and Zernicka-Goetz, 2014) (Figures 1A–1C, top). These cells did not display any sign of apoptosis after 24 h (Figure 1A, top), and only 13% of the structures contained cells positive for cleaved caspase-3 at 48 h (Figure 1B, top). Opening of the central lumen after 72 h was associated with the presence of apoptotic cells on the apical side in 50% of the structures examined (Figure 1C, top). In the absence of integrin β1, following cre-mediated excision of the *Itgβ1* locus (*Δ/Δ*), cells appeared indistinguishable from wild-type controls at 24 h, with only 2% of the integrin-deficient structures positive for cleaved caspase-3 (Figure 1A, bottom). Nevertheless, after 48 h, cells failed to form an organized rosette-like structure and 83% of the colonies displayed evident signs of apoptotic cell death on the basal side (Figure 1B, bottom). Eventually, after 72 h, all structures became positive for cleaved caspase-3, leading to lethality of the entire culture (Figure 1C, bottom, 100% penetrance). Despite the induction of apoptosis, the ability of cells to undergo proliferation was largely unaffected by the absence of integrin β1, as shown by the similar frequency of mitotic divisions between mutants and controls (Figures S1A–S1C). These results suggest that integrin β1 is required for the survival of mESCs, with loss of integrin β1 inducing the activation of apoptosis and ultimately leading to lethality. This is supported by a similar phenotype observed in human embryonic stem cells (hESCs), where integrin β1 is blocked (Kallas-Kivi et al., 2018; Vitillo et al., 2016).

To test whether integrin β1 was also necessary for the survival of the embryonic lineage *in vivo*, we generated mouse chimeras by the aggregation of wild-type embryos at 8- to 16-cell stage with the conditional mESC line carrying the *Itgβ1* locus (either *fl/fl* or *Δ/Δ*) (Figure S1D). Following blastocyst formation at embryonic day (E) E3.5, embryos were transferred into pseudo-pregnant surrogate females and recovered after formation of the post-implantation egg cylinder at E5.5 (Figure S1D). This system ensured confinement of the *Itgβ1* locus to the embryonic lineage only (epiblast, *Itgβ1 fl/fl* or *Δ/Δ*), without affecting the extra-embryonic tissues, such as the extraembryonic ectoderm (ExE; *Itgβ1* +/+) and the visceral endoderm (VE; *Itgβ1* +/+) (Figure S1D). When recovered at E5.5 (Figures 1D–1G), only 44% of embryos deficient for integrin β1 (Epi: *Itgβ1 Δ/Δ*) contained a clear epiblast compartment (Figures 1E and 1G), while the remaining 56% either lacked the epiblast completely or showed few residual $OCT4^+$ cells (Figures 1F and 1G). These results point toward an *in vivo* requirement of integrin β1 for the survival of the epiblast during post-implantation transition.

Figure 1. Integrin β1 is necessary for epiblast cell survival upon implantation and pluripotency transition

(A–C) Analysis of apoptosis by cleaved caspase-3 staining in wild-type (fl/fl, top) and mutant (Δ/Δ, bottom) mESC spheroids at 24 (A), 48 (B), and 72 h (C) of culture. Steady increase in the number of c-caspase-3^+ structures in mutants over time. Fisher's exact test: (A) p = ns (number of spheroids n = 42 [fl/fl], n = 44 [Δ/Δ]); (B) ****$p < 0.0001$ (n = 38 [fl/fl], n = 48 [Δ/Δ]); and (C) ****$p < 0.0001$ (n = 26 [fl/fl], n = 28 [Δ/Δ]).

(D–G) *In vivo*-recovered post-implantation embryos at E5.5, as shown in Figure S1D: epiblast is either fl/fl or Δ/Δ; visceral endoderm (VE) and extraembryonic ectoderm (ExE) are +/+. In contrast to wild-type embryos (Epi:fl/fl) (D), embryos deficient for integrin β1 (Epi:Δ/Δ) either have a small epiblast (E), show few residual $Oct4^+$ cells (F), or lack epiblast completely. Quantification of the number of embryos with epiblast versus degenerated/lethal—Fisher's exact test: ***p = 0.0003 (number of embryos n = 15 [fl/fl], n = 25 [Δ/Δ], 4 replicates).

(H–J) Assessment of apoptotic cells in chimeric blastocysts at E4.5–4.75, Epi:fl/fl (H, top) or Epi:Δ/Δ (H, bottom): embryos are sequentially re-stained to assess NANOG cells in the c-caspase-3 channel. Mutant embryos (Epi:Δ/Δ) contain a significantly higher number of apoptotic cells within the epiblast compartment: quantification of the percentage of c-caspase-3^+ cells over total epiblast cell number (I), and percentage of apoptotic cells in wild type (median = 1.7%) versus mutants (median = 14%), Mann-Whitney test; ****$p < 0.0001$ (number of embryos n = 37 [fl/fl], n = 29 [Δ/Δ]). Correlation between the percentage of c-caspase-3^+ cells with the percentage of either OTX2 (top) or $NANOG^+$ cells (bottom) within the epiblast (J): exit from naive pluripotency (increase in OTX2 and decrease in NANOG cells) correlates with the increase in the percentage of c-caspase-3^+ cells (number of embryos n = 23 [fl/fl], n = 15 [Δ/Δ]).

(K and L) Wild-type and mutant mESCs display comparable minimal levels of apoptotic cells when cultured in 2iLIF (naive pluripotency status, absence of OTX2 expression). (K) Fisher's exact test: p = ns (number of spheroids n = 33 [fl/fl], n = 37 [Δ/Δ]). In the absence of 2iLIF (L), cells transit to formative pluripotency as shown by the expression of OTX2 and activate the apoptotic pathway in the absence of integrin β1 (Δ/Δ).

Scale bars: 5 μm (A), 10 μm (B, C, K, and L), 15 μm (H, inset), 25 μm (H, inset, and D–F).

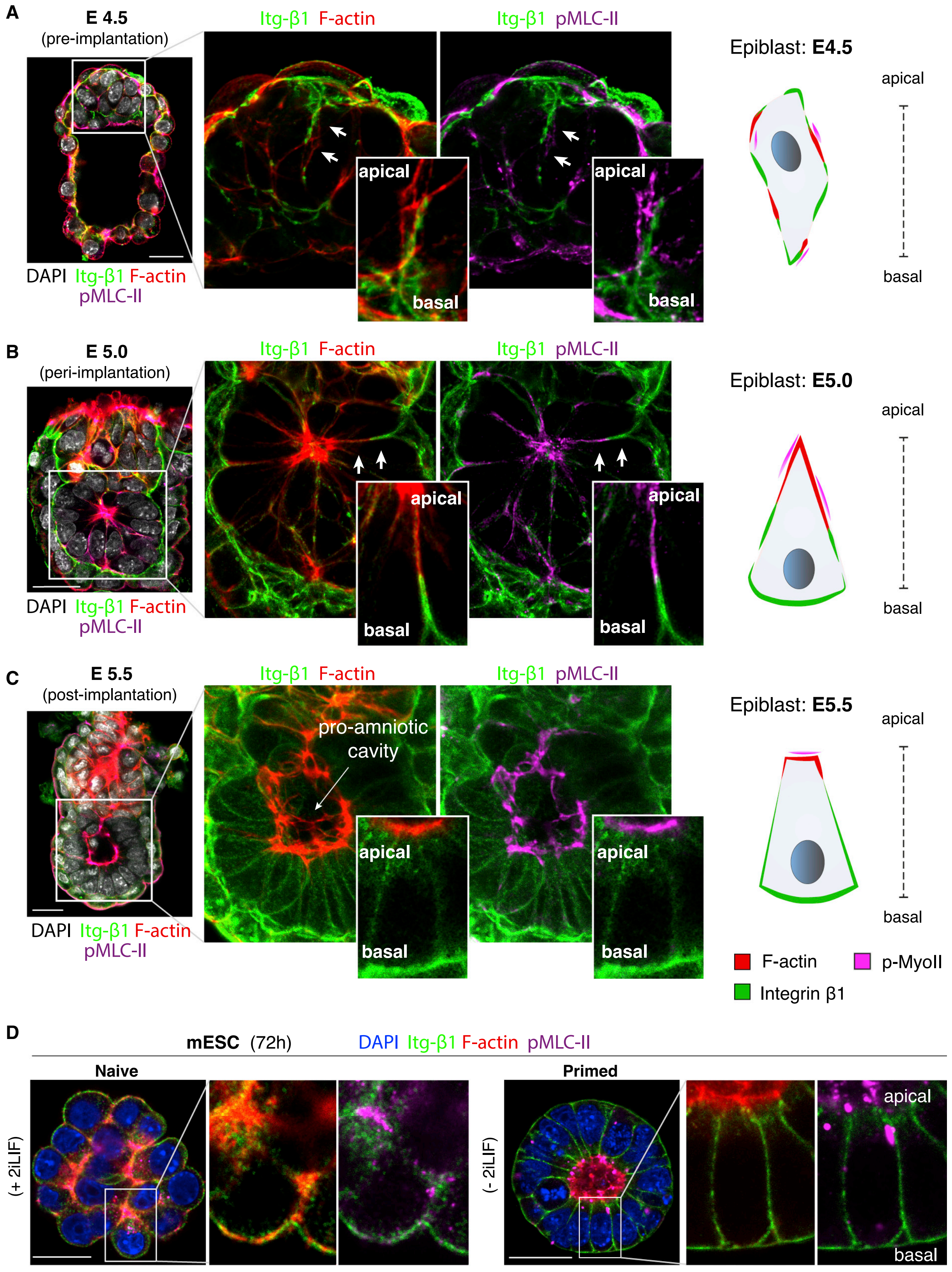

(legend on next page)

CellPress
OPEN ACCESS

To understand whether the observed phenotype was apparent during pre-implantation, we analyzed embryos at the late blastocyst stage (E4.5–E4.75) (Figure S1E). Despite normal pre-implantation development (Fässler and Meyer, 1995; Stephens et al., 1995), integrin β1-deficient embryos already displayed signs of apoptotic cell death within the epiblast compartment at E4.5 (Figure 1H). On average, 2%–3% of epiblast cells were positive for cleaved caspase-3 in the wild type as opposed to 14%–15% in the integrin β1 mutants (Figures 1I and S1F). Interestingly, initiation of apoptosis closely correlated with the exit from naive pluripotency and progression toward a formative state shown by the concomitant increase in OTX2 and reduction of NANOG expression (Figures 1H and 1J). This result suggests a time-specific dependency on integrin β1 for survival of the epiblast cells, becoming necessary as the epiblast cells exit from naive pluripotency and transit toward the formative state, as supported by the change in cell surface receptors between the two states in hESCs (Wojdyla et al., 2020).

To test whether integrin β1 was necessary for survival during naive pluripotency, we cultured mESC in 2iLIF (Kalkan and Smith, 2014; Nichols and Smith, 2012), thus preserving the expression of the naive marker NANOG. Surprisingly, only minimal levels of apoptotic cell death were observed in wild-type and mutant cells, regardless of the presence of integrin β1 (Figure 1K). In contrast, upon exit from naive pluripotency and transition toward the formative state, integrin-deficient cells became highly susceptible to cell death, as illustrated by the induction of apoptosis concomitant with the upregulation of OTX2, after 48 h of culture in differentiating conditions (−2iLIF) (Figure 1L). This reveals a time dependency of integrin-mediated signaling for survival of the embryonic lineage. Integrin β1 appears dispensable during naive pluripotency equivalent to early pre-implantation development (Figure S1G, left) while it becomes crucial upon the initiation of implantation and transition to the post-implantation formative state (Figure S1G, right).

In addition to survival, loss of integrin β1 impaired the ability of epiblast cells to arrange into a structured rosette configuration and form the mature post-implantation epithelium both *in vivo* (Figure 1E) and *in vitro* (Figures 1B and 1C) (Bedzhov and Zernicka-Goetz, 2014). This is an essential morphogenetic step required for the formation of the epiblast cavity to ensure successful post-implantation development (Bedzhov and Zernicka-Goetz, 2014; Luckett, 1975; Molè et al., 2020; Wallingford et al., 2013). By comparing sequential stages of mouse development from pre- to post-implantation, we observed a progressive spatial separation between integrin β1 and the contractile actomyosin cytoskeleton (Figure 2), central downstream target of the integrin signaling cascade (Harburger and Calderwood, 2009). While initially disorganized at E4.5 (Figure 2A), integrin β1 became segregated from the F-actin and phosphorylated non-muscle myosin (pMLC-II) at E5.0, when the epiblast forms an epithelium (Figure 2B, inset). At E5.5, this sorting process resulted in basolateral localization of integrin β1 and apical clustering of actomyosin (Figure 2C). This exact spatial segregation could be recapitulated *in vitro* with a complete separation between integrin β1 and actomyosin after 72 h of culture (Figure 2D, right), similar to the post-implantation epiblast epithelium. This peculiar patterning and segregation behavior led us to hypothesize that integrin β1 may exert an inhibitory action toward actomyosin by suppressing its assembly along the basal domain of the cells, at the site where integrins interact with the basement membrane.

To test this hypothesis, we analyzed the effect of integrin β1 removal on the spatial localization of the actomyosin cytoskeleton using mESC spheroids as a model (Figures 3A and 3B). Wild-type cells, carrying the unrecombined allele (*Itgβ1 fl/fl),* localized activated actomyosin at a central point along the cell-cell interface after 24 h of culture (Figure 3A, top), reminiscent of the apical membrane initiation site (AMIS) previously described in Madin-Darby canine kidney (MDCK) cells (Bryant et al., 2010, 2014). After 48 h, cells adopted a wedge-shaped morphology around the site of actomyosin enrichment, opening a central lumen (Figure 3B, top). In the absence of integrin β1 (*Δ/Δ)*, actomyosin accumulated ectopically on the basal domain of the cells at 24 h (Figure 3A, bottom) and formed a thick actomyosin cable running along the basal side of the colonies following 48 h of culture (Figure 3B, bottom). Alongside actomyosin accumulation, we observed the appearance of basal blebs (Figures 3C and S2A; Videos S1 and S2), which indicates an overall increase in basal cortical tension experienced by the colony due to ectopic accumulation of actomyosin. These structures appeared to be rich in E-cadherin, suggesting the formation of ectopic adherens junctions on the basal domain, which could disrupt the pattern of cell-cell adhesion for the formation of a structured epithelium. These results indicate that integrin β1 prevents the activation of the actomyosin cytoskeleton on the basolateral side of mESCs. Loss of integrin β1 abolishes the ability of cells to suppress actomyosin, which causes ectopic accumulation basally, preventing both the establishment of the rosette configuration and opening of the central cavity.

Similarly, loss of integrin β1 *in vivo* resulted in the ectopic activation of actomyosin on the basal side of the epiblast

Figure 2. Fine-tune spatial segregation between integrin β1 and actomyosin

(A) Configuration of the mouse epiblast before implantation (E4.5): cells are apolar and display heterogenous localization of integrin β1 (Itg-β1) and actomyosin, as shown by the distribution of phalloidin (F-actin) and phosphorylated non-muscle myosin (pMLC-II).

(B) Configuration of the mouse epiblast upon implantation (E5.0): segregation in a mutually exclusive manner between integrin β1 and actomyosin while cells acquire a wedge-shape morphology.

(C) Configuration of the mouse epiblast at post-implantation (E5.5): cells of the mature epiblast epithelium are columnar in shape surrounding a central pro-amniotic cavity. Integrin β1 localizes on the basolateral domain and actomyosin on the apical side.

(D) Integrin β1 and actomyosin are heterogeneously distributed in mouse embryonic stem cells (mESCs) maintained in naive pluripotency (+2iLIF, left panel), similarly to E4.5. Integrin β1 and actomyosin become spatially segregated during formative pluripotency following 72 h of removal of 2iLIF (−2iLIF, right panel), similarly to E5.5.

Scale bars: 25 μm (A–D). Magnified/inset areas indicated by arrows (A and B).

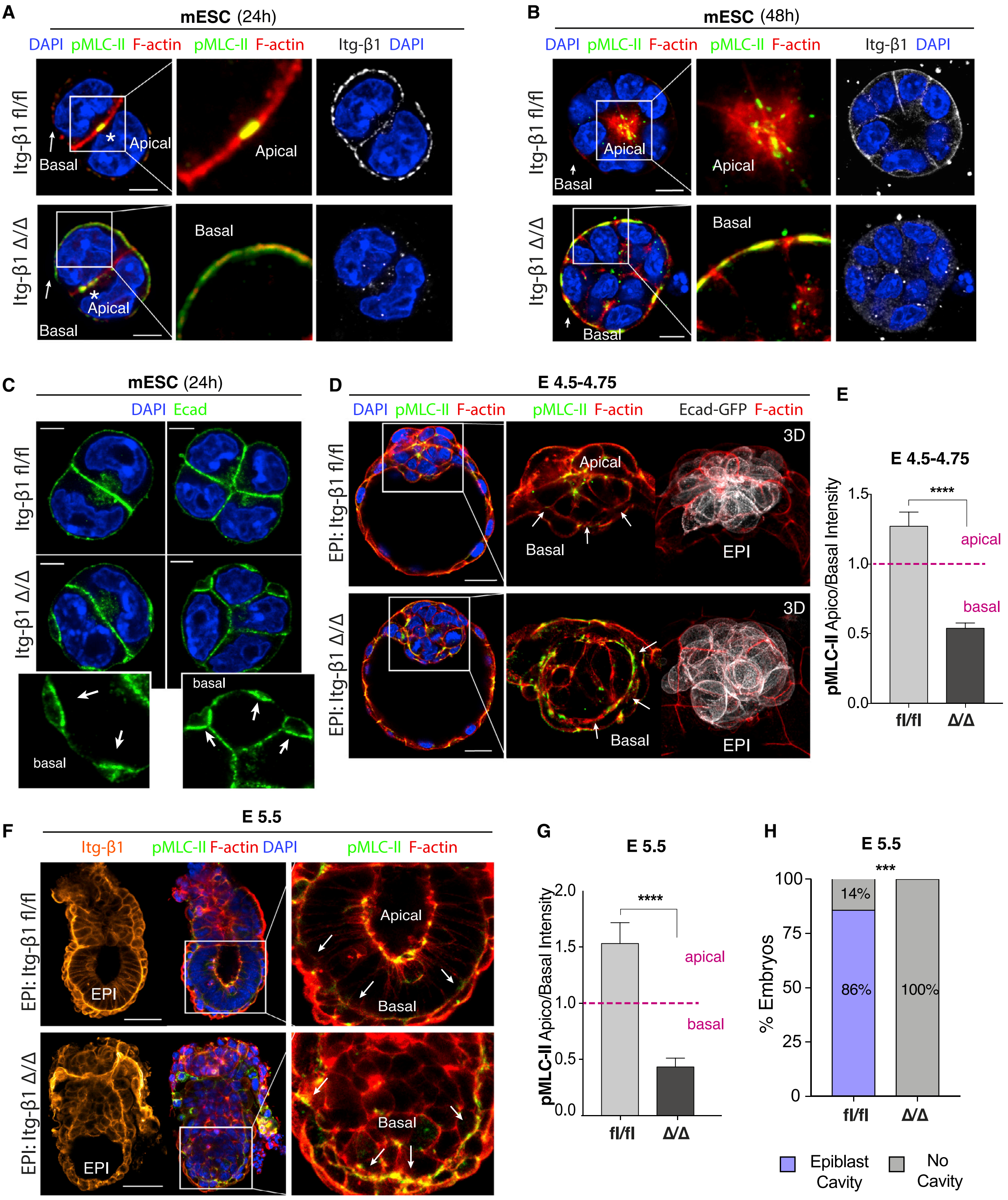

Figure 3. Loss of integrin β1 causes basal actomyosin accumulation and morphogenesis failure

(A and B) Distribution of actomyosin in mESC after 24 (A) and 48 h of culture (B). Wild-type cells (fl/fl) display enrichment of F-actin and p-myosin at the central point of the apical membrane initiation site (AMIS) at 24 h (A, top). Apical actomyosin is maintained after 48 h at the center of the rosette configuration (B, top). Mutant cells (Δ/Δ) display ectopic accumulation of actomyosin basally (A, bottom), giving rise to a large basal actomyosin cable after 48 h of culture (B, bottom).

(legend continued on next page)

(Figures 3D and 3E), prohibiting formation of the rosette-like configuration at the peri-implantation stage E4.5–E4.75. In the presence of integrin β1, cells of the epiblast were able to successfully adopt a wedge-shaped morphology and form a 3D rosette structure around a central point of apical actomyosin accumulation (Figure 3D, top). To assess the effect of integrin β1 loss and ectopic actomyosin activation on morphogenesis of the post-implantation epiblast, chimeric embryos were transferred into surrogate mothers and recovered after formation of the post-implantation egg cylinder, as before (Figure S1D). Integrin β1-deficient embryos activated actomyosin on the basal side of the epiblast (Figures 3F and 3G), failing to give rise to a fully structured epiblast epithelium and to open the central lumen at E5.5 (Figure 3H). Thus, integrin β1 localization along the basolateral domain of epiblast cells prevents the assembly of actomyosin upon the initiation of implantation. This local inhibition ensures the confinement of actomyosin on the apical domain, which is an essential prerequisite for the formation of the rosette and opening of the lumen in the center of the epiblast epithelium. While recruitment of integrins has usually been associated with the stimulation of actin polymerization leading to the formation of stress fibers, focal adhesion, lamellipodia, and filipodia (Huttenlocher and Horwitz, 2011; Kechagia et al., 2019; Mitra et al., 2005), integrins appear to act as suppressors of the contractile cytoskeleton during these early stages of embryonic development.

We then asked how failure to suppress actomyosin on the basolateral domain of cells could prevent epiblast morphogenesis. Numerous studies have recognized the importance of integrins for the establishment of apicobasal polarity in different epithelial models (Akhtar and Streuli, 2013; Aumailley et al., 2000; Li et al., 2017; Yu et al., 2005). However, we found that despite the loss of integrin β1, mESCs were able to localize the apical polarity marker PAR3 (Figures 4A and 4B), the Golgi apparatus (Figure S2D), and the centrosomes (Figures 4C and 4D) toward the incipient apical initiation site at 24 h. Even in the absence of any extracellular matrix components, when plating cells in agarose, cells deficient for integrin β1 were capable of initiating establishment of the apicobasal polarity (Figures 4E and 4F). However, following 48 h of culture, integrin β1-deficient cells were not able to maintain their apicobasal polarity, while wild-type cells developed a clear apical domain demarcated by both PAR3 and PAR6 (Figures 4G–4I and S2E). The inability to maintain polarity at this stage was not caused by defects in the surrounding extracellular matrix (ECM), as shown by the ability of both wild-type and mutant cells to assemble a fully structured basement membrane on their basal domains (Figures S2B and S2C). Instead, this phenotype appeared to be caused by defects in the localization of PAR6 and recruitment of podocalyxin vesicles. While largely absent at 24 h (Figure S2D), PAR6 became expressed after 48 h of culture at the apical side of the cells, localizing at the site of actomyosin accumulation (Figures 4G and 4H). Instead, in integrin-deficient cells, PAR6 was recruited toward the basal domain, which exhibited high actomyosin accumulation (Figure 4G). While in the wild-type podocalyxin vesicles were directed toward the apically localized PAR6 (Figure 4H, top) facilitating opening of the lumen, podocalyxin vesicles were directed toward the basally localized PAR6 in the absence of integrin β1 (Figure 4H, bottom). This prevented their secretion in the center of the epithelium for the initiation of lumenogenesis. This result suggests that integrin β1 may be dispensable for the initial establishment of polarity but becomes necessary for its maintenance.

Hence, our results indicate that integrin β1 is involved in promoting epiblast survival by the suppression of apoptosis and in regulating epithelial fate by actomyosin repression. To determine whether we could rescue pharmacologically the lethality caused by integrin β1 deficiency via mimicking its suppressive action toward actomyosin, we targeted the Rho-associated kinase ROCK, as a major downstream target of integrin signaling (Huveneers and Danen, 2009). Similarly, ROCK inhibition has been shown to prevent apoptosis and augment lumenogenesis in human ESCs (Ohgushi and Sasai, 2011; Taniguchi et al., 2015; Watanabe et al., 2007). The supplementation of the ROCK inhibitor Y27632 in the culture medium was able to abolish the ectopic basal actomyosin accumulation, inducing mutant cells to re-adopt a rosette-like configuration similar to controls (Figure 5A), as observed in MDCK cells (Yu et al., 2008). Cells were able to restore apical localization of the atypical protein kinase C (aPKC) and podocalyxin secretion at this site, thus rescuing lumenogenesis (Figure 5A). However, ROCK inhibition failed to prevent the induction of apoptosis. Despite the re-establishment of apical polarity, 77% of the ESC colonies were positive for cleaved caspase-3 (Figure 5B), suggesting a second mechanism beyond actomyosin remodeling for the regulation of epiblast survival.

Two major pathways downstream of integrins have been shown to promote cell survival, the AKT and the ERK pathways (Moreno-Layseca and Streuli, 2014). Signaling from growth factors is integrated downstream of integrins to promote cell-cycle progression, protecting cells from a type of cell death called

(C) Blebs and ectopic E-cadherin junctions appear on the basal domain of mutant mESCs (arrows).

(D) Distribution of actomyosin in the mouse blastocyst at E4.5, chimeric for either fl/fl (top) or Δ/Δ (bottom) alleles of *Itgβ1*. Wild-type epiblasts (Epi:fl/fl) accumulate actomyosin at the central point of apical constriction, forming a rosette configuration (top). Mutant epiblasts (Epi:Δ/Δ) accumulate actomyosin basally and fail to form the rosette (bottom).

(E) Fluorescence intensity quantification of apicobasal p-myosin (pMLC-II) at E4.5 shows significant increase of basal myosin in mutant epiblasts. Fluorescence intensity = mean ± SEM, Mann-Whitney test: ****$p < 0.0001$ (number of embryos n = 21 [fl/fl], n = 23 [Δ/Δ]).

(F) Assessment of actomyosin localization in post-implantation embryos at E5.5. Wild-type epiblasts (Epi:fl/fl) accumulate actomyosin at the apical side of the epiblast epithelium. Mutant epiblasts (Epi:Δ/Δ) accumulate actomyosin basally and fail to form the central pro-amniotic cavity.

(G) Fluorescence intensity quantification of apicobasal p-myosin (pMLC-II) at E5.5: significant increase in basal myosin in mutant epiblasts. Fluorescence intensity = mean ± SEM, Mann-Whitney test: ****$p < 0.0001$ (number of embryos n = 14 [fl/fl], n = 8 [Δ/Δ]).

(H) Quantification of percentage of embryos forming cavity in the epiblast at E5.5: all mutant epiblasts fail to form the cavity: Fisher's exact test: ****$p = 0.0001$ (number of embryos n = 14 [fl/fl], n = 8 [Δ/Δ]).

Scale bars: 5 μm (A), 10 μm (B and C), 25 μm (D), and 50 μm (F).

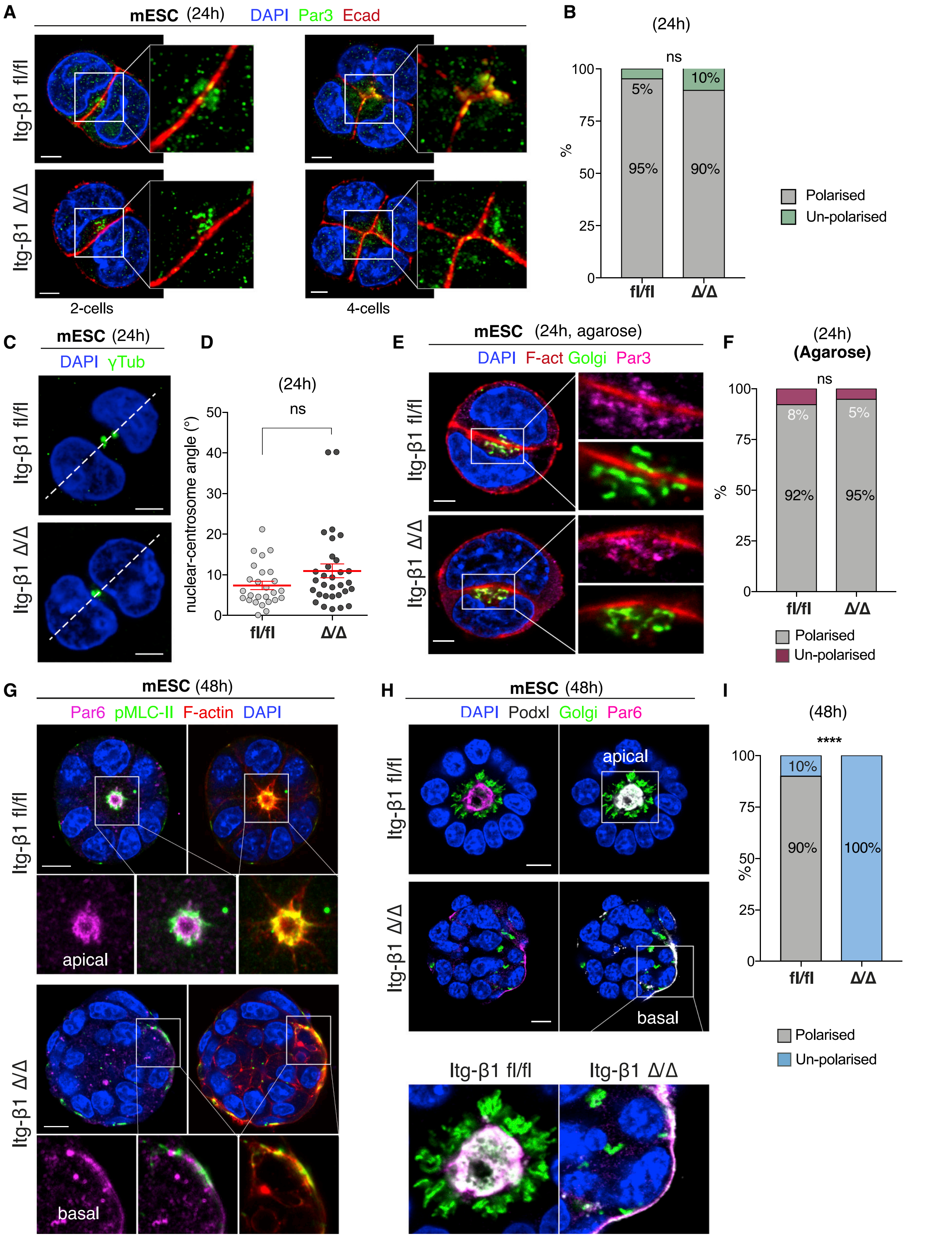

(legend on next page)

anoikis, which results from the loss of integrin-mediated adhesion (Chiarugi and Giannoni, 2008). Thus, we tested whether pharmacological supplementation of insulin-like growth factor 1 (IGF1) and glycogen synthase kinase 3 inhibitor (GSK3i) to activate AKT (Denduluri et al., 2015) and fibroblast growth factor 2 (FGF2) to stimulate ERK/MAPK (mitogen-activated protein kinase) (Lanner and Rossant, 2010) could rescue lethality. We tested whether supplementation of FGF2 alone (Figure S3A), IGF1 together with GSK3i (Figure S3C), or the combination of all 3 growth factors (FGF2, IGF1, and GSK3i) (Figure 5C) could rescue the epiblast lethality. However, after 48 h of culture, ESC colonies deficient for integrin β1 were still positive for cleaved caspase-3 as opposed to wild-type controls. We then tested the combination of FGF2 (Figure S3B) or IGF1/GSK3i (Figure S3D) while supplementing ROCK inhibitor as well. Once again, colonies deficient for integrin β1 still displayed cleaved caspase-3 compared to wild type. However, when the stimulation of pro-survival signals by FGF2, IGF1, and GSK3i was coupled with actomyosin inhibition by ROCKi, both survival and morphogenesis could be restored fully despite integrin β1 deficiency (Figures 5D and S3E), resulting in comparable levels of apoptotic structures between wild type and mutant. Furthermore, the ectopic accumulation of actomyosin on the basal domain of integrin β1-deficient cells (Figures 5E and 5F) was suppressed and actomyosin localization restored at the apical domain, promoting the initiation of lumen formation (Figures 5G and 5H). These results suggest that both the contractile cytoskeleton and the pro-survival signals need to be coordinated to promote survival and morphogenesis of the epiblast cells. Survival cannot be induced without simultaneous stabilization of the cytoskeleton and restoration of morphogenesis.

To assess whether a similar mechanism occurs *in vivo*, we cultured chimeric embryos beyond implantation in the presence of the above factors using a modified *in vitro* culture system (Ma et al., 2019) (Figure S3F). While embryos deficient for integrin β1 failed to undergo lumenogenesis and degenerated (Figure 6A, center), as we previously observed (Figures 1E–1G), pharmacological suppression of ROCK together with the supplementation of FGF2, IGF1, and GSK3i was able to fully rescue the lethality induced by the loss of integrin β1, promoting formation of the lumen in the epiblast and the egg cylinder formation to a comparable level to wild-type controls (Figures 5A and 5B). This confirmed that suppression of basal actomyosin via ROCK inhibition, together with the stimulation by FGF2, IGF1, and GSK3i, are sufficient also *in vivo* to induce normal morphogenesis and survival of the epiblast during its transition from pre- to post-implantation, despite integrin β1 deficiency.

DISCUSSION

In this study, we describe a role for integrin β1 in survival and morphogenesis of the epiblast during the pre- to post-implantation transition of the mouse embryo (Figure 6C). Suppression of the contractile cytoskeleton on the basolateral domain of the epiblast epithelium by the action of integrin β1 ensures the confinement of actomyosin at the apical domain (Figure 6C, left). This is necessary for the maturation of the epiblast epithelium and the opening of the central lumen, a mechanism apparently conserved in both mouse and human embryos (Figure 6D). Loss of integrin β1 prevents suppression of the cytoskeleton basally, leading to ectopic accumulation of actomyosin fibers on the basal side of the epiblast (Figure 6C, right). A similar phenotype has been observed in embryoid bodies lacking core components of the integrin adhesome complex (Horton et al., 2016), such as ILK (Sakai et al., 2003) and PINCH1 (Li et al., 2005), leading ultimately to the failure of the formation of a structured epithelium. Nonetheless, ectopic actomyosin and hypercontractility at the basal side of the epiblast can be counteracted by the inhibition of ROCK as shown in MDCK cells (Bryant et al., 2014; Yu et al., 2008), pharmacologically mimicking the inhibitory effect of integrin-mediated adhesion on the contractile cytoskeleton. Actomyosin in the apical domain appears to be largely unaffected by ROCK inhibition, suggesting an additional mechanism for the activation of actomyosin apically, independent of integrin β1. A likely candidate is Cdc42-MRCK, which has been shown to regulate apical actomyosin recruitment in the MDCK model (Zihni et al., 2017).

The basal actomyosin accumulation observed in integrin β1-deficient cells differs from the inverted polarity phenotype described in MDCK cells (Bryant et al., 2014; Davis and Cleaver, 2014; Yu et al., 2005) and mammary gland epithelia (Akhtar and Streuli, 2013). We find that mESCs are capable of initiating apicobasal polarization despite the loss of integrin β1, even in the absence of any ECM components. This indicates that cell-ECM interactions are dispensable for the initiation of polarization, which has been recently observed during morphogenesis of the follicular epithelium in the *Drosophila* egg chamber (Lovegrove et al., 2019). However, the maintenance of polarity is dependent on integrin β1

Figure 4. Integrin β1 is dispensable for the establishment but necessary for the maintenance of apicobasal polarity

(A) Distribution of the polarity marker PAR3 in mESCs at 1- and 2-cell stage (24 h of culture in −2iLIF). Wild-type cells (fl/fl, top) recruit PAR3 toward the AMIS similarly to mutant cells (Δ/Δ, bottom).

(B) Quantification of the orientation of apicobasal polarity at 24 h: Fisher's exact test: p = ns (number of mESC spheroids n = 42 [fl/fl], n = 39 [Δ/Δ]).

(C and D) Distribution of centrosomes, as shown by γ-tubulin staining, at 2-cell stage and quantification of the angles along the nuclear-centrosome axis. Centrosome-nuclear axis angle: means ± SEMs (red). Test: Mann-Whitney test: p = ns (number of mESC spheroids n = 26 [fl/fl], n = 31 [Δ/Δ]).

(E and F) Assessment of polarization in agarose at 24 h by Golgi and PAR3 localization. Fisher's exact test: p = ns (number of spheroids n = 38 [fl/fl], 38 [Δ/Δ]). Both wild-type and mutant cells display correct apicobasal polarity at 24 h of culture, even in the absence of ECM components.

(G) Assessment of polarization at 48 h. PAR6 is recruited at the apical site where actomyosin accumulated in wild type. PAR6 is recruited basally at the site of actomyosin accumulation in mutant cells.

(H) In wild type, podocalyxin vesicles are recruited apically at PAR6 site. In mutant, PAR6 localizes basally, and podocalyxin vesicles are secreted basally at this latter site.

(I) Quantification of the orientation of apicobasal polarity at 48 h, assessed by Golgi and PAR6 localization. Fisher's exact test: ****p < 0.0001 (n = 30 [fl/fl], n = 29 [Δ/Δ]). Scale bars: 5 μm (A, C, and E) and 10 μm (G and H).

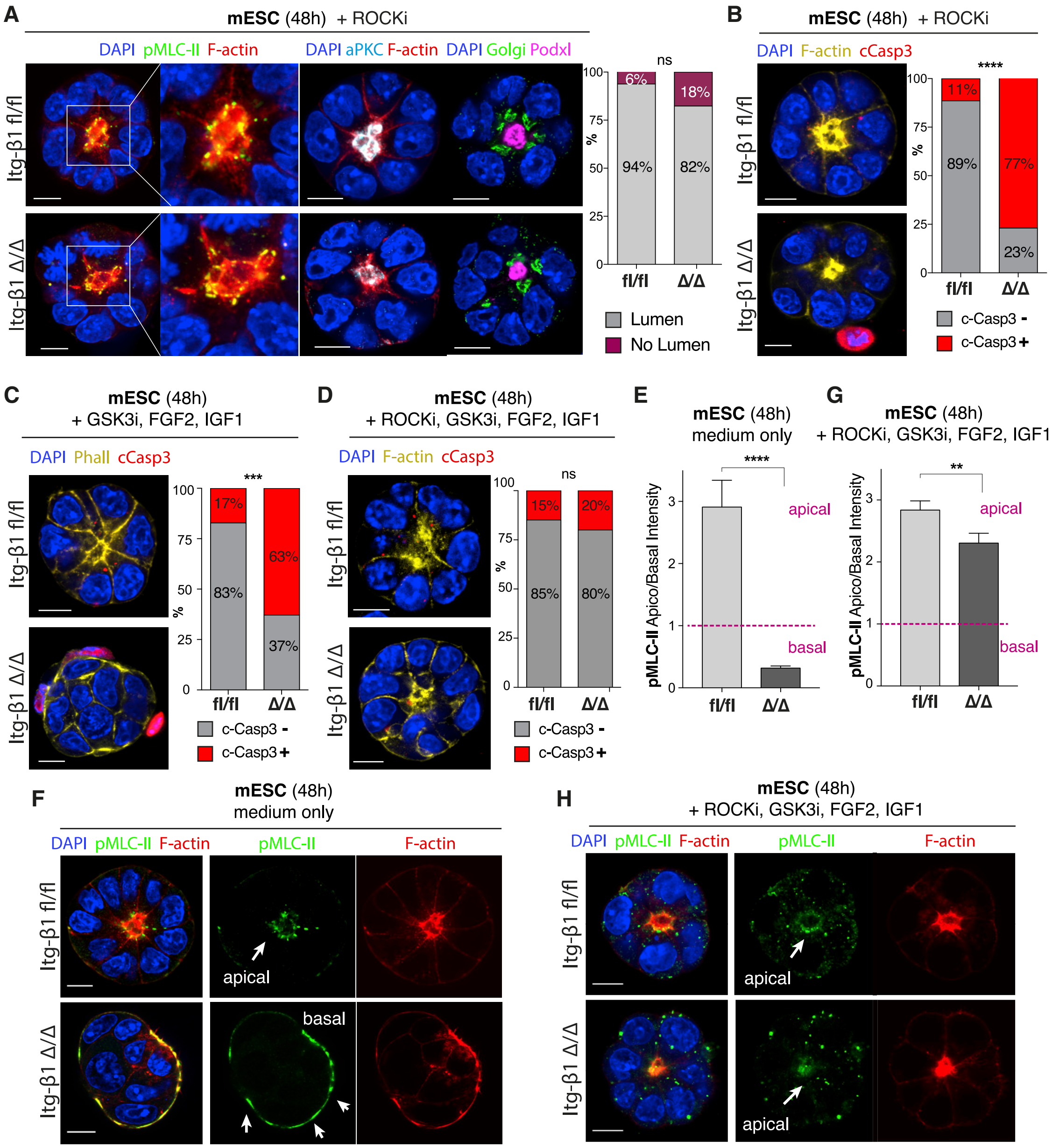

Figure 5. *In vitro* rescue of integrin-mediated survival and morphogenesis via FGF/IGF stimulation and ROCK inhibition

(A) ROCK inhibition by Y27632 rescues lumen initiation in mESCs deficient for integrin β1 (Δ/Δ, bottom): Golgi is correctly oriented apically, podocalyxin vesicles are secreted apically, polarity marker aPKC is recruited at the apical domain, and actomyosin is re-established apically. Quantification of the percentage of mESC spheroids undergoing lumenogenesis shows no significant differences between wild-type and mutant cells: Fisher's exact test: p = ns (number of spheroids n = 65 [fl/fl], n = 68 [Δ/Δ]).

(B) Assessment of structures showing apoptotic cell death following ROCK inhibition treatment: mutant cells activate apoptosis despite rescue of polarity and lumen formation. Fisher's exact test: ****$p < 0.0001$ (number of spheroids n = 35 [fl/fl], n = 39 [Δ/Δ]).

(C) Stimulation of the survival pathways via supplementation of FGF2, IGF1, and GSK3i is not sufficient to prevent initiation of apoptosis in integrin β1 mutant cells (Δ/Δ): Fisher's exact test: ***p = 0.0002 (n = number of spheroids = 35 [fl/fl], n = 35 [Δ/Δ]).

(D) Inhibition of ROCK coupled to supplementation of FGF2, IGF1, and GSK3i restores both morphogenesis and survival in spheroids deficient for integrin β1 (Δ/Δ, bottom): Fisher's exact test: p = ns (number of spheroids n = 47 [fl/fl], n = 40 [Δ/Δ]).

(E and F) Fluorescence intensity quantification of apicobasal p-myosin (pMLC-II) in mESCs spheroids cultured for 48 h in medium only shows myosin ectopic localization on the basal side of mutant cells (ratio <1). Fluorescence intensity = mean ± SEM, Mann-Whitney test: ****$p < 0.0001$ (number of spheroids n = 31 [fl/fl], n = 30 [Δ/Δ]).

(G and H) Fluorescence intensity quantification of apicobasal p-myosin (pMLC-II) in mESCs spheroids supplemented with FGF2, IGF1, GSK3i, and ROCKi for 48 h shows re-establishment of myosin in the apical domain in mutant cells (ratio >1). Fluorescence intensity = mean ± SEM, Mann-Whitney test: **p = 0.002 (number of spheroids n = 35 [fl/fl], n = 31 [Δ/Δ]). Despite the increase in the ratio of apicobasal p-myosin, mutants still differ significantly from wild types.

Scale bars: 10 μm (A–D, F, and H),

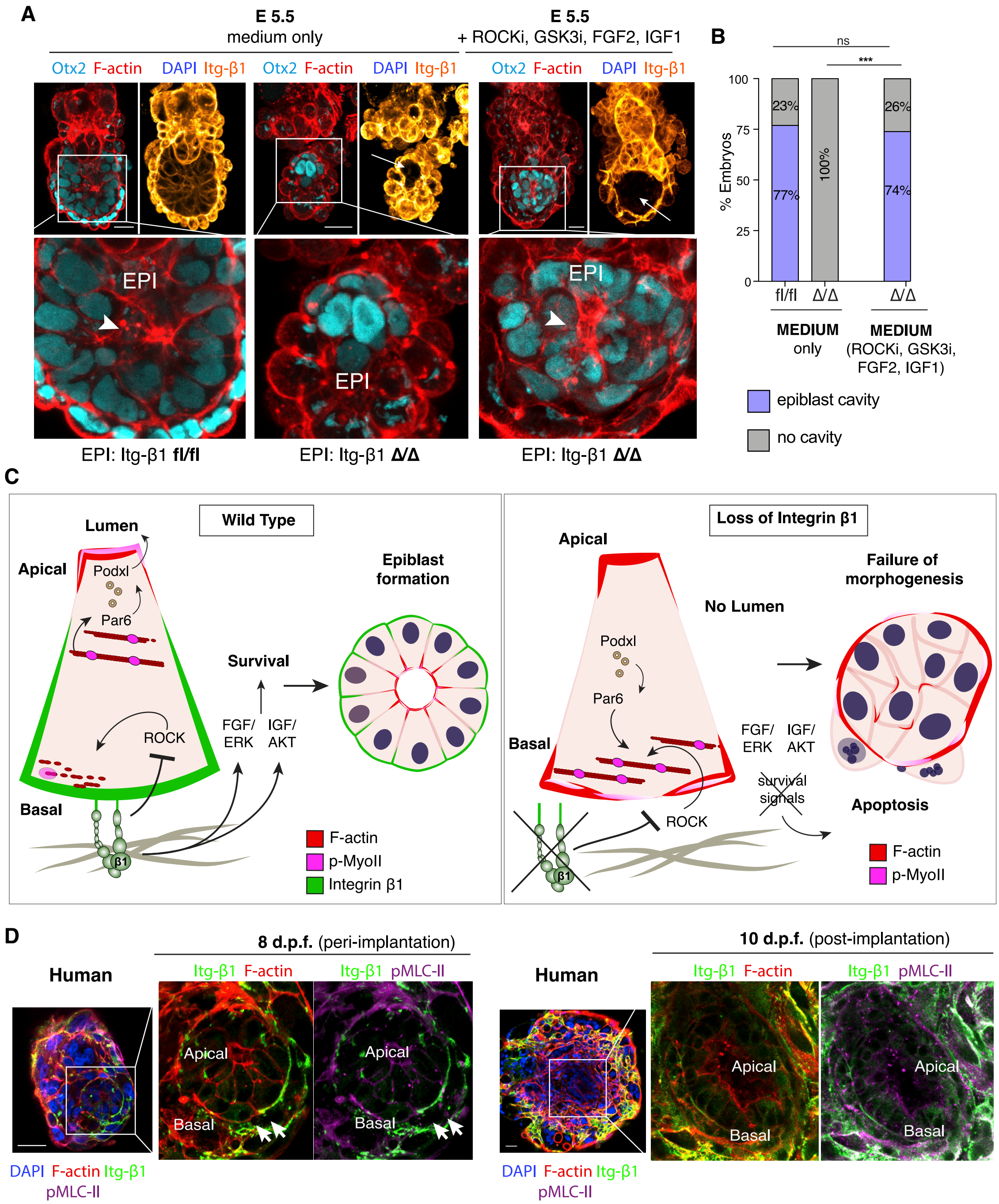

Figure 6. *In vivo* rescue of survival and morphogenesis in mouse embryos and spatial segregation of integrin β1 and actomyosin in the human embryo

(A) Culture of mouse embryos from pre- to post-implantation as shown in Figure S3F: wild-type controls (Epi:fl/fl) develop into post-implantation egg-cylinders (left), mutant epiblasts deficient for integrin β1 (Epi:Δ/Δ) fail to undergo lumenogenesis and to survive during post-implantation development in normal culture conditions (center). Supplementation with ROCKi, FGF2, IGF1, and GSK3i restores lumenogenesis and survival of the epiblast compartment in integrin β1-deficient embryos (right).

(B) Quantification of the percentage of embryos undergoing lumenogenesis in wild-type and mutant embryos cultured in normal conditions compared to mutant embryos cultured in the presence of ROCKi, FGF2, IGF1, and GSK3i. Fisher's exact test: fl/fl versus Δ/Δ ROCKi/FGF2/IGF1/GSK3i p = ns; Δ/Δ medium versus Δ/Δ ROCKi/FGF2/IGF1/GSK3i ****p < 0.0001 (number of embryos n = 26 [fl/fl], n = 16 [Δ/Δ], n = 51 [Δ/Δ, ROCKi/FGF2/IGF1/GSK3i]).

(C) Schematic summary of results. Wild-type (left): integrin β1-mediated adhesion to the basement membrane leads to suppression of actomyosin basally, allowing its apical localization. Activation of actomyosin at the apical side leads to the apical localization of PAR6 (Figure 4G) and secretion of podocalyxin vesicles

(legend continued on next page)

due to integrin-mediated regulation of actomyosin. Loss of this inhibitory effect mediated by integrins and the resulting ectopic activation of actomyosin basally appears to prevail on the apical activation of actomyosin. The polarity complex PAR6-aPCK appears to be highly sensitive to asymmetries in cortical tension and has been shown to be transported by the actomyosin flow (Goehring et al., 2011; Mayer et al., 2010; Munro et al., 2004). In the wild-type situation, actomyosin activation in the apical domain causes PAR6 recruitment at this site (Figure 6C, left). Podocalyxin vescicles are secreted at the site of PAR6 localization, causing expansion of the lumen apically. Conversely, in the absence of integrin β1, PAR6 is recruited basally to the site of actomyosin accumulation (Figure 6C, right). In turn, podocalyxin vesicles are directed toward this site, preventing their secretion in the center of the epiblast for the initiation of lumenogenesis. This results in an overall loss of epithelial polarity rather than acquisition of an inverted polarity phenotype.

Our results indicate that alongside cytoskeletal remodeling, the presence of integrin β1 is also important for survival of the epiblast (Figure 6C, left). While dispensable during pre-implantation development and naive pluripotency, its action becomes necessary upon transition toward formative/primed pluripotency, when cells acquire mechanosensitivity to physical stresses (Verstreken et al., 2019). The time-specific induction of apoptosis closely parallels the lethality observed in the integrin β1 knockout (KO) mouse that occurs following implantation (Fässler and Meyer, 1995; Stephens et al., 1995), highlighting a requirement of integrin-mediated signals for epiblast survival upon implantation.

Survival by supplementation of FGF2 and IGF1/GSK3i could not be rescued independently of the stabilization of the actomyosin cytoskeleton, suggesting that survival and morphogenesis must be tightly coupled (Figure 6C, left). Similarly, stabilization of the cytoskeleton alone does not prevent the induction of cell death, suggesting that the rescue of morphogenesis *per se* is not sufficient to promote survival of the epiblast. These results indicate that integrins coordinate both survival and maturation of the epiblast epithelium for transition of the embryo to post-implantation development.

A similar mechanism of actomyosin spatial segregation seems to be conserved in the human embryo (Figure 6D). Similar to the mouse, integrin β1 and the actomyosin cytoskeleton become progressively and mutually segregated following transition of the human embryo through implantation, 8 days post-fertilization (d.p.f.) (Figure 6D, left). As the embryo develops into post-implantation (10 d.p.f.), integrin β1 adopts a basolateral localization, while actomyosin becomes confined to the apical site, where the amniotic cavity forms (Figure 6D, right). This supports the possibility that integrin β1 may also be important in the human embryo, providing insights into potential molecular requirements for successful human embryogenesis during this critical stage of development.

STAR★METHODS

Detailed methods are provided in the online version of this paper and include the following:

- KEY RESOURCES TABLE
- RESOURCE AVAILABILITY
  - Lead contact
  - Materials availability
  - Data and code availability
- EXPERIMENTAL MODEL AND SUBJECT DETAILS
  - Mice
  - Mouse embryonic stem cells (mESC)
  - Human Embryos
- METHOD DETAILS
  - Mouse embryo recovery
  - Mouse embryo culture media
  - Chimera and mouse embryo culture
  - Human Embryo Culture
  - Mouse embryonic stem cells media and supplements
  - Derivation of mouse embryonic stem cells
  - Propagation of mouse embryonic stem cells
  - 3D culture of mouse embryonic stem cells
  - Fixation
  - Immunofluorescence staining
  - Time-lapse movies
- QUANTIFICATION AND STATISTICAL ANALYSIS
  - Image acquisition
  - Quantifications
  - Statistical analysis

SUPPLEMENTAL INFORMATION

Supplemental Information can be found online at https://doi.org/10.1016/j.celrep.2021.108834.

ACKNOWLEDGMENTS

We thank all of the members of the M.Z.-G. lab for critical discussion. We are grateful to all of the patients for donating their embryos and embryologists at the CARE clinics for help and support. We thank Marta N. Shahbazi for her help in thawing the human embryos. We thank William Mansfield for the support with mouse embryos transfers. Work in the M.Z.-G. laboratory is supported by grants from the Wellcome Trust (207415/Z/17/Z); the ERC (grant no. 669198); EU Horizon 2020 Marie Skłodowska-Curie actions (ImageInLife, 721537); the NIH (R01 HD100456-01A1); the NIH Pioneer Award Fund (DP1 HD104575-01); the Open Philanthropy/Silicon Valley Community Foundation; the Weston Havens Foundation; and the Shurl and Kay Curci Foundation, to M.Z.-G.

and initiation of lumenogenesis (Figure 4H). Integrin β1 promotes epiblast survival via stimulation of FGF/ERK and IGF1/AKT pathways. Mutant (right): loss of integrin β1 leads to ectopic actomyosin accumulation basally. The basal actomyosin recruits PAR6 basally, which directs podocalyxin vesicles toward the basal side, preventing lumenogenesis. Loss of integrin β1 leads to apoptosis.

(D) Assessment of the localization of integrin β1 and actomyosin in human embryos at day 8 d.p.f. shows initiation of spatial segregation between the 2 complexes in a mutually exclusive manner (arrows). At 10 d.p.f., the 2 are fully segregated, with integrin β1 localizing on the basolateral domain, while actomyosin is confined on the apical side of the epiblast epithelium facing the central amniotic cavity.

Scale bars: 25 μm (A) and 50 μm (D).

AUTHOR CONTRIBUTIONS

M.A.M. and M.Z.-G. conceptualized and designed the project. M.A.M. and A.W. performed and analyzed the experiments regarding mESCs and mouse embryos. M.A.M. carried out the experiments regarding human embryos. A.W. and M.A.M. prepared the illustrations. A.C. and S.F. oversaw and provided human embryos for these studies. R.F. provided the conditional integrin β1 mouse line. M.Z.-G. supervised the project. M.A.M. wrote the manuscript with the help of M.Z.-G.

DECLARATION OF INTERESTS

The authors declare no competing interests.

Received: May 12, 2020
Revised: November 30, 2020
Accepted: February 17, 2021
Published: March 9, 2021

REFERENCES

Akhtar, N., and Streuli, C.H. (2013). An integrin-ILK-microtubule network orients cell polarity and lumen formation in glandular epithelium. Nat. Cell Biol. *15*, 17–27.

Aplin, J.D., and Ruane, P.T. (2017). Embryo-epithelium interactions during implantation at a glance. J. Cell Sci. *130*, 15–22.

Aumailley, M., Pesch, M., Tunggal, L., Gaill, F., and Fässler, R. (2000). Altered synthesis of laminin 1 and absence of basement membrane component deposition in (beta)1 integrin-deficient embryoid bodies. J. Cell Sci. *113*, 259–268.

Barczyk, M., Carracedo, S., and Gullberg, D. (2010). Integrins. Cell Tissue Res. *339*, 269–280.

Bedzhov, I., and Zernicka-Goetz, M. (2014). Self-organizing properties of mouse pluripotent cells initiate morphogenesis upon implantation. Cell *156*, 1032–1044.

Boroviak, T., and Nichols, J. (2017). Primate embryogenesis predicts the hallmarks of human naïve pluripotency. Development *144*, 175–186.

Bryant, D.M., Datta, A., Rodríguez-Fraticelli, A.E., Peränen, J., Martín-Belmonte, F., and Mostov, K.E. (2010). A molecular network for de novo generation of the apical surface and lumen. Nat. Cell Biol. *12*, 1035–1045.

Bryant, D.M.M., Roignot, J., Datta, A., Overeem, A.W.W., Kim, M., Yu, W., Peng, X., Eastburn, D.J.J., Ewald, A.J.J., Werb, Z., and Mostov, K.E. (2014). A molecular switch for the orientation of epithelial cell polarization. Dev. Cell *31*, 171–187.

Cakmak, H., and Taylor, H.S. (2011). Implantation failure: molecular mechanisms and clinical treatment. Hum. Reprod. Update *17*, 242–253.

Campbell, I.D., and Humphries, M.J. (2011). Integrin structure, activation, and interactions. Cold Spring Harb. Perspect. Biol. *3*, 369–397.

Cha, J., Sun, X., and Dey, S.K. (2012). Mechanisms of implantation: strategies for successful pregnancy. Nat. Med. *18*, 1754–1767.

Chiarugi, P., and Giannoni, E. (2008). Anoikis: a necessary death program for anchorage-dependent cells. Biochem. Pharmacol. *76*, 1352–1364.

Davis, G.E., and Cleaver, O.B. (2014). Outside in: inversion of cell polarity controls epithelial lumen formation. Dev. Cell *31*, 140–142.

Denduluri, S.K., Idowu, O., Wang, Z., Liao, Z., Yan, Z., Mohammed, M.K., Ye, J., Wei, Q., Wang, J., Zhao, L., and Luu, H.H. (2015). Insulin-like growth factor (IGF) signaling in tumorigenesis and the development of cancer drug resistance. Genes Dis. *2*, 13–25.

Fässler, R., and Meyer, M. (1995). Consequences of lack of beta 1 integrin gene expression in mice. Genes Dev. *9*, 1896–1908.

Geisert, R.D., and Fuller, W.B. (2015). Regulation of Implantation and Establishment of Pregnancy in Mammals (Springer).

Goehring, N.W., Trong, P.K., Bois, J.S., Chowdhury, D., Nicola, E.M., Hyman, A.A., and Grill, S.W. (2011). Polarization of PAR Proteins by Advective Triggering of a Pattern-Forming System. Science *334*, 1137–1141.

Hackett, J.A., and Surani, M.A. (2014). Regulatory principles of pluripotency: from the ground state up. Cell Stem Cell *15*, 416–430.

Harburger, D.S., and Calderwood, D.A. (2009). Integrin signalling at a glance. J. Cell Sci. *122*, 159–163.

Horton, E.R., Humphries, J.D., James, J., Jones, M.C., Askari, J.A., and Humphries, M.J. (2016). The integrin adhesome network at a glance. J. Cell Sci. *129*, 4159–4163.

Huttenlocher, A., and Horwitz, A.R. (2011). Integrins in cell migration. Cold Spring Harb. Perspect. Biol. *3*, a005074.

Huveneers, S., and Danen, E.H.J. (2009). Adhesion signaling - crosstalk between integrins, Src and Rho. J. Cell Sci. *122*, 1059–1069.

Kalkan, T., and Smith, A. (2014). Mapping the route from naive pluripotency to lineage specification. Philos. Trans. R. Soc. Lond. B Biol. Sci. *369*, 20130540.

Kallas-Kivi, A., Trei, A., Stepanjuk, A., Ruisu, K., Kask, K., Pooga, M., and Maimets, T. (2018). The role of integrin β1 in the heterogeneity of human embryonic stem cells culture. Biol. Open *7*, bio034355.

Kechagia, J.Z., Ivaska, J., and Roca-Cusachs, P. (2019). Integrins as biomechanical sensors of the microenvironment. Nat. Rev. Mol. Cell Biol. *20*, 457–473.

Kinoshita, M., Barber, M., Mansfield, W., Cui, Y., Spindlow, D., Stirparo, G.G., Dietmann, S., Nichols, J., and Smith, A. (2020). Capture of Mouse and Human Stem Cells with Features of Formative Pluripotency. Cell Stem Cell, S1934-5909(20)30543-9.

Koot, Y.E.M., Teklenburg, G., Salker, M.S., Brosens, J.J., and Macklon, N.S. (2012). Molecular aspects of implantation failure. Biochim. Biophys. Acta *1822*, 1943–1950.

Lanner, F., and Rossant, J. (2010). The role of FGF/Erk signaling in pluripotent cells. Development *137*, 3351–3360.

Li, S., Bordoy, R., Stanchi, F., Moser, M., Braun, A., Kudlacek, O., Wewer, U.M., Yurchenco, P.D., and Fässler, R. (2005). PINCH1 regulates cell-matrix and cell-cell adhesions, cell polarity and cell survival during the peri-implantation stage. J. Cell Sci. *118*, 2913–2921.

Li, S., Qi, Y., McKee, K., Liu, J., Hsu, J., and Yurchenco, P.D. (2017). Integrin and dystroglycan compensate each other to mediate laminin-dependent basement membrane assembly and epiblast polarization. Matrix Biol. *57-58*, 272–284.

Lovegrove, H.E., Bergstralh, D.T., and St Johnston, D. (2019). The role of integrins in *Drosophila* egg chamber morphogenesis. Development *146*, 706069.

Lucas, E.S., Dyer, N.P., Fishwick, K., Ott, S., and Brosens, J.J. (2016). Success after failure: the role of endometrial stem cells in recurrent miscarriage. Reproduction *152*, R159–R166.

Luckett, W.P. (1975). The development of primordial and definitive amniotic cavities in early Rhesus monkey and human embryos. Am. J. Anat. *144*, 149–167.

Ma, H., Zhai, J., Wan, H., Jiang, X., Wang, X., Wang, L., Xiang, Y., He, X., Zhao, Z.-A., Zhao, B., et al. (2019). In vitro culture of cynomolgus monkey embryos beyond early gastrulation. Science *366*, eaax7890.

Macklon, N.S., Geraedts, J.P.M., and Fauser, B.C.J.M. (2002). Conception to ongoing pregnancy: the 'black box' of early pregnancy loss. Hum. Reprod. Update *8*, 333–343.

Mayer, M., Depken, M., Bois, J.S., Jülicher, F., and Grill, S.W. (2010). Anisotropies in cortical tension reveal the physical basis of polarizing cortical flows. Nature *467*, 617–621.

Miner, J.H., Li, C., Mudd, J.L., Go, G., and Sutherland, A.E. (2004). Compositional and structural requirements for laminin and basement membranes during mouse embryo implantation and gastrulation. Development *131*, 2247–2256.

Mitra, S.K., Hanson, D.A., and Schlaepfer, D.D. (2005). Focal adhesion kinase: in command and control of cell motility. Nat. Rev. Mol. Cell Biol. *6*, 56–68.

Molè, M.A., Weberling, A., and Zernicka-Goetz, M. (2020). Comparative analysis of human and mouse development: from zygote to pre-gastrulation. Curr. Top. Dev. Biol. *136*, 113–138.

Moore, R., Tao, W., Smith, E.R., and Xu, X.-X. (2014). The primitive endoderm segregates from the epiblast in β1 integrin-deficient early mouse embryos. Mol. Cell. Biol. *34*, 560–572.

Moreno-Layseca, P., and Streuli, C.H. (2014). Signalling pathways linking integrins with cell cycle progression. Matrix Biol. *34*, 144–153.

Mulas, C., Kalkan, T., von Meyenn, F., Leitch, H.G., Nichols, J., and Smith, A. (2019). Defined conditions for propagation and manipulation of mouse embryonic stem cells. Development *146*, dev173146.

Munro, E., Nance, J., and Priess, J.R. (2004). Cortical flows powered by asymmetrical contraction transport PAR proteins to establish and maintain anterior-posterior polarity in the early C. elegans embryo. Dev. Cell *7*, 413–424.

Nichols, J., and Smith, A. (2012). Pluripotency in the embryo and in culture. Cold Spring Harb. Perspect. Biol. *4*, a008128.

Norwitz, E.R., Schust, D.J., and Fisher, S.J. (2001). Implantation and the survival of early pregnancy. N. Engl. J. Med. *345*, 1400–1408.

Ohgushi, M., and Sasai, Y. (2011). Lonely death dance of human pluripotent stem cells: ROCKing between metastable cell states. Trends Cell Biol. *21*, 274–282.

Potocnik, A.J., Brakebusch, C., and Fässler, R. (2000). Fetal and adult hematopoietic stem cells require beta1 integrin function for colonizing fetal liver, spleen, and bone marrow. Immunity *12*, 653–663.

Sakai, T., Li, S., Docheva, D., Grashoff, C., Sakai, K., Kostka, G., Braun, A., Pfeifer, A., Yurchenco, P.D., and Fässler, R. (2003). Integrin-linked kinase (ILK) is required for polarizing the epiblast, cell adhesion, and controlling actin accumulation. Genes Dev. *17*, 926–940.

Schindelin, J., Arganda-Carreras, I., Frise, E., Kaynig, V., Longair, M., Pietzsch, T., Preibisch, S., Rueden, C., Saalfeld, S., Schmid, B., et al. (2012). Fiji: an open-source platform for biological-image analysis. Nat. Methods *9*, 676–682.

Schwartz, M.A. (2010). Integrins and extracellular matrix in mechanotransduction. Cold Spring Harb. Perspect. Biol. *2*, a005066.

Shahbazi, M.N., and Zernicka-Goetz, M. (2018). Deconstructing and reconstructing the mouse and human early embryo. Nat. Cell Biol. *20*, 878–887.

Smyth, N., Vatansever, H.S., Murray, P., Meyer, M., Frie, C., Paulsson, M., and Edgar, D. (1999). Absence of basement membranes after targeting the LAMC1 gene results in embryonic lethality due to failure of endoderm differentiation. J. Cell Biol. *144*, 151–160.

Stephens, L.E., Sutherland, A.E., Klimanskaya, I.V., Andrieux, A., Meneses, J., Pedersen, R.A., and Damsky, C.H. (1995). Deletion of beta 1 integrins in mice results in inner cell mass failure and peri-implantation lethality. Genes Dev. *9*, 1883–1895.

Sutherland, A.E., Calarco, P.G., and Damsky, C.H. (1993). Developmental regulation of integrin expression at the time of implantation in the mouse embryo. Development *119*, 1175–1186.

Taniguchi, K., Shao, Y., Townshend, R.F., Tsai, Y.-H., DeLong, C.J., Lopez, S.A., Gayen, S., Freddo, A.M., Chue, D.J., Thomas, D.J., et al. (2015). Lumen Formation Is an Intrinsic Property of Isolated Human Pluripotent Stem Cells. Stem Cell Reports *5*, 954–962.

Verstreken, C.M., Labouesse, C., Agley, C.C., and Chalut, K.J. (2019). Embryonic stem cells become mechanoresponsive upon exit from ground state of pluripotency. Open Biol. *9*, 180203.

Vitillo, L., Baxter, M., Iskender, B., Whiting, P., and Kimber, S.J. (2016). Integrin-Associated Focal Adhesion Kinase Protects Human Embryonic Stem Cells from Apoptosis, Detachment, and Differentiation. Stem Cell Reports *7*, 167–176.

Wallingford, M.C., Angelo, J.R., and Mager, J. (2013). Morphogenetic analysis of peri-implantation development. Dev. Dyn. *242*, 1110–1120.

Watanabe, K., Ueno, M., Kamiya, D., Nishiyama, A., Matsumura, M., Wataya, T., Takahashi, J.B., Nishikawa, S., Nishikawa, S., Muguruma, K., and Sasai, Y. (2007). A ROCK inhibitor permits survival of dissociated human embryonic stem cells. Nat. Biotechnol. *25*, 681–686.

Wilcox, A.J., Weinberg, C.R., O'Connor, J.F., Baird, D.D., Schlatterer, J.P., Canfield, R.E., Armstrong, E.G., and Nisula, B.C. (1988). Incidence of early loss of pregnancy. N. Engl. J. Med. *319*, 189–194.

Wilcox, A.J., Baird, D.D., and Weinberg, C.R. (1999). Time of implantation of the conceptus and loss of pregnancy. N. Engl. J. Med. *340*, 1796–1799.

Wojdyla, K., Collier, A.J., Fabian, C., Nisi, P.S., Biggins, L., Oxley, D., and Rugg-Gunn, P.J. (2020). Cell-Surface Proteomics Identifies Differences in Signaling and Adhesion Protein Expression between Naive and Primed Human Pluripotent Stem Cells. Stem Cell Reports *14*, 972–988.

Wu, J., Yamauchi, T., and Izpisua Belmonte, J.C. (2016). An overview of mammalian pluripotency. Development *143*, 1644–1648.

Yu, W., Datta, A., Leroy, P., O'Brien, L.E., Mak, G., Jou, T.-S., Matlin, K.S., Mostov, K.E., and Zegers, M.M.P. (2005). Beta1-integrin orients epithelial polarity via Rac1 and laminin. Mol. Biol. Cell *16*, 433–445.

Yu, W., Shewan, A.M., Brakeman, P., Eastburn, D.J., Datta, A., Bryant, D.M., Fan, Q.-W., Weiss, W.A., Zegers, M.M.P., and Mostov, K.E. (2008). Involvement of RhoA, ROCK I and myosin II in inverted orientation of epithelial polarity. EMBO Rep. *9*, 923–929.

Zihni, C., Vlassaks, E., Terry, S., Carlton, J., Leung, T.K.C., Olson, M., Pichaud, F., Balda, M.S., and Matter, K. (2017). An apical MRCK-driven morphogenetic pathway controls epithelial polarity. Nat. Cell Biol. *19*, 1049–1060.

STAR★METHODS

KEY RESOURCES TABLE

REAGENT or RESOURCE	SOURCE	IDENTIFIER
Antibodies		
Integrin β1 (for mouse embryos and mESCs)	Merck Millipore	MAB1997, RRID: AB_2128202
Integrin β1 (for human embryos)	Merck Millipore	MABT821
pMLC-II	Cell Signaling	3671S, RRID: AB_330248
Par3	Merck Millipore	07-330, RRID: AB_2101325
Par6	Santa Cruz	SC-67393, RRID: AB_2267889
E-cadherin	Thermo Fisher Scientific	13-1900, RRID: AB_86571
Podocalyxin	R&D Systems	MAB1556, RRID: AB_2166010
Golgi (GM130)	BD Biosciences	BD610822, RRID: AB_398141
γ-Tubulin	Sigma	T6557, RRID: AB_477584
Cleaved-Caspase 3	Cell Signaling	9664, D175, 5A1E, RRID: AB_2070042
Otx2	R&D Systems	AF1979, RRID: AB_2157172
GFP	Nacalai USA	GF090R, RRID: AB_10013361
PKC	Santa Cruz	SC-17781, RRID: AB_628148
Laminins	Sigma-Merck	L9393, RRID: AB_477163
Perlecan	Merck	MAB1948P, RRID: AB_10615958
Oct3/4	Santa Cruz	sc-5279, RRID: AB_628051
Nanog	Abcam	ab80892, RRID: AB_2150114
pHH3	Merck Millipore	06-570, RRID: AB_310177
Chemicals, peptides, and recombinant proteins		
B27	Thermo Fisher Scientific	17504001
N2	Home-made - MZG Lab, Thermo Fisher Scientific	17502048
Y27632	StemCell Technologies, Inc.	72304
PD0325901	Stem Cell Institute, Cambridge	N/A
GSK3 inhibitor	Stem Cell Institute Cambridge	N/A
LIF	Stem Cell Institute Cambridge	N/A
TrypLE Express Enzyme	Thermo Fisher Scientific	12604-021
Matrigel	SLS	354230
IGF1	Stem Cell Technologies	78078
FGF2 zebrafish	Stem Cell Institute Cambridge	N/A
Experimental models: cell lines		
Mouse Embryonic Stem Cells	This manuscript	N/A
Experimental models: organisms/strains		
Mice: wild type CD1	Charles River Strain code: 022	Strain code: 022
Mice: wild type F1	Charles River	Strain Code 176
Mice: Itgb1^{tm1Ref} (Integrin β1 floxed)	Potocnik et al., 2000	MGI: 1926498
Human embryos	This manuscript	N/A
Oligonucleotides Itgββ1 PCR genotyping floxed versus wild type allele		
Forward: CTTTGCGTTGTCAGCATGGG	This manuscript	N/A
Reverse: ACACTGCCATCTGCCTTTCT	This manuscript	N/A
Recombinant DNA		
pRN3p-E-cadherin-eGFP	MZG lab	N/A

(*Continued on next page*)

Continued

REAGENT or RESOURCE	SOURCE	IDENTIFIER
Software and algorithms		
Prism GraphPad 8	N/A	https://www.graphpad.com/scientific-software/prism/
Fiji (ImageJ)	N/A	https://fiji.sc/
Adobe Illustrator CC	N/A	https://www.adobe.com/uk/products/illustrator.html

RESOURCE AVAILABILITY

Lead contact

Further information and requests for resources and reagents should be directed to and will be fulfilled by the Lead Contact, Magdalena Zernicka-Goetz (mz205@cam.ac.uk).

Materials availability

All unique/stable reagents generated in this study are available from the Lead Contact with a completed Materials Transfer Agreement.

Data and code availability

This study did not generate any unique datasets or code.

EXPERIMENTAL MODEL AND SUBJECT DETAILS

Mice

Mice were bred in the Gurdon Institute Animal House. All animal studies were performed according to the regulations of the UK Animals (Scientific Procedures) Act 1986 and the Medical Research Council's Responsibility in the Use of Animals for Medical Research (July 1993), following ethical review by the University of Cambridge Animal Welfare and Ethical Review Body (AWERB) and approval by the Home Office. Animals were kept in a pathogen-free facility and housed in individually ventilated cages (IVCs). Mice used for the experiments are F1 wild-type (C57BL/6 × CBA) naturally mated aged 3-4 months, except for superovulation procedures where females aged 5-6 weeks. Superovulation was performed by injection of 7.5 IU of pregnant mares' serum gonadotropin (PMSG, Intervet), followed by injection of 7.5 IU of human chorionic gonadotropin (hCG, Intervet) after 48h and mating. Mice were time-mated overnight and checked the following morning for the presence of a copulation plug, designated as embryonic day E0.5.

The mouse line for the floxed allele of integrin-β1 (Potocnik et al., 2000) was a gift of Reinhard Fässler (gene symbol: Itgb1^{tm1Ref}, MGI: 1926498) and used to generate the integrin β1 mESC conditional lines: homozygous *fl/fl* (floxed unrecombined, wild-type) and *Δ/Δ* (deleted, mutant). The genotype of the mice was determined by PCR on DNA samples extracted from ear-clips using the following primers: CTTTGCGTTGTCAGCATGGG and ACACTGCCATCTGCCTTTCT. PCR cycles were the following: 95°C 3 min; 35 cycles x 95°C 30 s, 53°C 30 s, 72°C 1 min; 72°C 5 min. Band products: 500 bp (floxed allele), 300 bp (wild-type allele).

Mouse embryonic stem cells (mESC)

Integrin β1 *fl/fl* mESCs (wild-type) were derived from pre-implantation mouse embryos (Mulas et al., 2019) by mating between mice homozygous for the floxed *Itgβ1* allele (*Itgβ1*$^{fl/fl}$). Cells were assessed for the expression of core pluripotency markers OCT4, NANOG and SOX2 and maintained in 2iLIF conditions (see below). The genotype was confirmed by PCR. Integrin β1 *Δ/Δ* mESC (mutant) was obtained by cre-mediated recombination of *Itgβ1* floxed allele as explained in the Method details section below. Ecad-GFP was introduced by stable transfection as shown in the Method details section below.

Human Embryos

Human embryo work was done in accordance with Human Fertilization and Embryology Authority (HFEA) regulations (license reference R0193). Ethical approval was obtained from the "Human Biology Research Ethics Committee" of the University of Cambridge (HBREC.2017.21). Informed consent was obtained from all participants in the study. These were patients from CARE Fertility Group clinics, which donated surplus embryos after completing IVF treatment. Prior to giving consent, patients were informed about the specific objectives of the project, and the conditions that apply within the license, offered counselling, and did not receive any financial inducements. Embryos were donated at day 5 stage (d.p.f). Cultures were stopped before day 14 and prior to the appearance of any signs of primitive streak formation according to the international 14 day rule.

METHOD DETAILS

Mouse embryo recovery

Pregnant females were killed by cervical dislocation and the uterine horns, including oviducts and ovaries, were explanted and dissected in M2 medium. Embryos at E2.5 (8-16 cell stage) were recovered by flushing of the oviducts from super-ovulated females, *zona pellucida* was removed by treatment with acidic tyrode's solution (T1788, Sigma) and embryos were used for chimeric aggregation experiments. Embryos at E4.5 were recovered by flushing the uterus of naturally mated females with M2 medium. Embryos at E5.0 and E5.5 were recovered from naturally mated females by dissection from the decidual tissue and manual removal of the Reichert's membrane.

Mouse embryo culture media

KSOM medium. KSOM base (MR-020P-5F, Millipore) supplemented with 1X essential amino acids (11130-036, Thermo Fisher Scientific), 1X non-essential amino acids (11140-035, Thermo Fisher Scientific) and 3 mM glucose (G8644, Sigma).

Medium for *in vitro* culture is a modification of the medium originally used for the culture of monkey embryo described in Ma et al. (2019): CMRL (11530037, Thermo Fisher Scientific) supplemented with 1X B27 (17504001, Thermo Fisher Scientific), 1X N2 (homemade or commercial 17502048, Thermo Fisher Scientific), 1X penicillin–streptomycin (15140122, Thermo Fisher Scientific), 1X GlutaMAX (35050-038, Thermo Fisher Scientific), 1X sodium pyruvate (11360039, Thermo Fisher Scientific), 1X essential amino acids (11130-036, Thermo Fisher Scientific), 1X non-essential amino acids (11140-035, Thermo Fisher Scientific), 1.8 mM glucose (G8644, Sigma). Various concentrations of Fetal Bovine Serum (Stem Cell Institute) are addended at different stages of cultures as shown in Figure S3E.

N2 homemade supplement. DMEM F12 medium, 2.5 mg/ml insulin (I9287, Sigma-Aldrich), 10 mg/ml Apo- transferrin (T1147, Sigma-Aldrich), 0.75% bovine albumin fraction V (15260037, Thermo Fisher Scientific), 20 μg/ml progesterone (p8783, Sigma-Aldrich), 1.6 mg/ml putrescine dihydrochloride (P5780, Sigma-Aldrich) and 6 μg/ml sodium selenite (S5261, Sigma-Aldrich).

Chimera and mouse embryo culture

Wild-Type embryos at E2.5, 8-16 cell stage (not compacted), were aggregated with either *Itgβ1 fl/fl* (wild-type) or *Δ/Δ* (mutant) mESCs, containing an overexpression GFP-tagged transgene *Ecad* to assess the levels of chimerism. Aggregated embryos were cultured in drops of KSOM medium for 24-hours at 37°C (5% CO_2) covered with mineral oil (Biocare Europe SRL, 9305) in a humidifier incubator.

For *in vivo* embryo transfer experiments presented in Figures 1D–1F and 3F, chimeric embryos at E3.5 stage (after 24-hours in KSOM as described above) were transferred to E2.5 pseudo-pregnant females that had been mated with vasectomised males. Embryos are then recovered 72-hours after, equivalent to E5.5 stage by dissection of decidual tissue (see Figure S1D). To note: extra-embryonic lineages (extraembryonic ectoderm and visceral endoderm) are derived from the wild-type embryo, while the epiblast compartment from mESCs homozygous for either the floxed (fl/fl) or deleted allele (Δ/Δ) of *Itgβ1*. This ensures confinement of the mutation only in the epiblast compartment, without affecting the ability of extraembryonic tissue to mediate implantation.

For *ex vivo* embryo culture experiments to the blastocyst stage E4.5-4.75 presented in Figures 1H and 3D, after 24-hours in KSOM, chimeric embryos were transferred into *in vitro* culture medium supplemented with 10% FBS for further 24-hours till reaching an expanded blastocyst state (see Figure S1E).

For *ex vivo* embryo culture rescue experiments to stage E5.5 presented in Figure 6A, please refer to Figure S3F. Initially 8-16 cell stage were aggregated with either *Itgβ1 fl/fl* (wild-type) or *Δ/Δ* (mutant) mESCs and cultured in drops of KSOM medium for 24-hours (time point: 0h). After KSOM, embryos were transferred in drops of *in vitro* culture medium supplemented with 10% FBS, ROCKi, FGF2, IGF1 and GSK3i for further 24-hours (time point: 24h). When reached the expanded blastocyst state (time point: 48h), embryos were transferred in 8-well μ-Slides in nylon mesh (150 μm, Plastok 03-150/50) and cultured in medium covered by oil with 20% FBS for further 24-hours and with 30% FBS for final 48-hours. Embryos attached to the side wall of the nylon mesh and remain suspended giving rise to the egg cylinder structures. After 48-hours culture in the nylon mesh (time point: 96h), embryos were manually dissected from the nylon mesh and incubated in drops of medium for final 24-hours. ROCK inhibitor (Y27632, Stem Cell Technology, 72304, resuspended in DMSO) was supplemented in medium at 20 μM for initial 48-hours and 40 μM for the final 48-hours as shown in Figure S3E. GSK3i at 0.75 μM (Chiron, CHIR99021, Stem Cell Institute Cambridge), FGF2 at 50 ng/ml (Stem Cell Institute Cambridge) and IGF1 at 100 ng/ml (Stem Cell 78078, resuspended in water) were supplemented in the medium for the total culture period.

Human Embryo Culture

Cryopreserved day 5 (d.p.f) human blastocysts were received at the University of Cambridge and thawed using the Kitazato thawing kit (VT802-2, Hunter Scientific) following the manufacturer's instructions as below. The day before thawing, the TS solution was placed at 37°C sealed and Global Total human embryo culture medium (HGGT-030, LifeGlobal group) was incubated at 37°C + 5% CO_2 overnight. Upon thawing, human embryos were immersed in 1mL of pre-warmed TS solution for 1 minute. Subsequently, they were transferred to DS solution for 3 minutes, WS1 solution for 5 minutes and WS2 solution for 1 minute. All these incubation steps were done using reproplates (REPROPLATE, Hunter Scientific). Thawed embryos were incubated in pre-equilibrated Global

Total human embryo culture medium covered with mineral oil (9305, Irvine Scientific) for 4h to allow recovery. Culture conditions were the following: 37°C 21% O_2 and 5% CO_2. After 4h incubation in Global, the *zona pellucida* was removed by brief treatment of the embryos with acidic tyrode's solution (T1788, Sigma). Embryos were subsequently cultured in pre-equilibrated *in vitro* culture 1 (IVC1) culture medium (M11-25, Cell Guidance Systems), supplemented with 50 ng/mL of Insulin Growth Factor 1 (IGF-1) (78078, STEMCELL Technologies). Embryos were cultured in 8 well μ-slides tissue culture treated (80826, Ibidi). Half-media changes were done every 24 hours. Embryos were finally rinsed in ice-cold phosphate buffered saline (PBS) and fixed in 4% PFA for 20 min.

Mouse embryonic stem cells media and supplements

FC medium. DMEM (41966 or 11995, Thermo Fisher Scientific), 15% Fetal Bovine Serum (Stem Cell Institute), 1X penicillin–streptomycin (15140122, Thermo Fisher Scientific), 1X GlutaMAX (35050-038, Thermo Fisher Scientific), 1X non-essential amino acids (11140-035, Thermo Fisher Scientific), 1X sodium pyruvate (11360039, Thermo Fisher Scientific) and 100 μM β-mercaptoethanol (31350-010, Thermo Fisher Scientific).

N2B27 medium is a mix of 50% DMEM F12 (21331-020, Thermo Fisher Scientific) and 50% of Neurobasal A (10888-022, Thermo Fisher Scientific), supplemented with 1X B27 (17504001, Thermo Fisher Scientific), 1X N2 (homemade or 17502048, Thermo Fisher Scientific), 100 μM β-mercaptoethanol (31350-010, Thermo Fisher Scientific), 1X penicillin–streptomycin (15140122, Thermo Fisher Scientific) and 1X 1X GlutaMAX (35050-038, Thermo Fisher Scientific).

2iLIF supplementation. 1 μM MEK inhibitor PD0325901 (Stem Cell Institute), 3 μM GSK3 inhibitor CHIR99021 (Stem Cell Institute) and 10 ng/ml LIF (Stem Cell Institute) was added to FC or N2B27 medium to preserve naive pluripotency.

Derivation of mouse embryonic stem cells

mESCs homozygous for integrin β1 (*fl/fl*) were generated as follows. Mice homozygous for the floxed *Itgβ1* allele (*Itgβ1*$^{fl/fl}$, gene symbol: Itgb1^{tm1Ref}, MGI: 1926498) were time-mated and homozygous *fl/fl* embryos were recovered at E2.5 (8-16 cell stage) from the oviducts. Embryos were cultured in KSOM (MR-020P-5F, Millipore) supplemented with 2iLIF for 24h at 37°C, 21% O_2 and 5% CO_2. Embryos were then incubated in N2B27 supplemented with 2iLIF for 48h. Hatched embryos were then individually plated in flat-bottomed 96-well plate (167008, Thermo Fisher Scientific) coated with inactivated mitomycin C-treated MEFs (GSC-6101G, Amsbio) in FC+2iLIF until a cell outgrowth was clearly visible. The outgrowth was then dissociated with 0.05% trypsin-EDTA (25300054, Thermo Fisher Scientific) and propagated in the absence of MEFs as shown below in the section entitled Propagation of Mouse Embryonic Stem Cells.

mESCs homozygous for integrin β1 (*Δ/Δ*) were generated as follows: mESCs homozygous for integrin β1 (*fl/fl*) were dissociated into single cells by trypsin treatment and cultured in FC medium supplemented with 2iLIF and 4 μM of TAT-cre recombinase (SCR508, Millipore) for 16h. Cre mediates recombination of the *Itgβ1* floxed allele (*fl*) into deleted allele (Δ). Recombined cells were selected single close isolation and by LacZ staining due to activation of a promoterless *LacZ* transgene inserted at the end of the *Itgβ1* locus. Briefly, cells were fixed in freshly prepared 0.2% glutaraldehyde solution. A lacZ solution was prepared as follows: 10 mM Potassium Ferrocyanide ($K_3Fe(CN)_6$, 10 mM Potassium Ferrocyanide ($K_4Fe(CN)_6$. 3 H_2O, 2 mM $MgCl_2$, 20 μL Nonidet P40 and 20 mM Tris HCl pH 7.5 in PBS. X-Gal (5-Bromo-4Chloro-3Indolyl-β-D- Galactopyranoside Sigma B4252) was dissolved in DMSO (dimethyl sulfoxide) to a concentration of 100 mg/ml of X-Gal in DMSO. This latter was eventually diluted in the lacZ solution to a final concentration of 1 mg/ml of X-Gal. LacZ solution with X-Gal was pre-warmed to 37°C to dissolve X-Gal and then passed through a 0.22 μm filter to eliminate any X-Gal precipitates. Cells were incubated at 37°C overnight, with protection from light. Blue clones (recombined) were isolated, propagated as single clones, and assessed for the lack of expression of integrin β1 by immunofluorescence.

mESCs Ecad-GFP / integrin β1 (*fl/fl*) and (*Δ/Δ*) were generated by stable transfection of the above lines as follows: Ecad-GFP sequence from pRN3p-E-cadherin-eGFP plasmid was first sub-cloned following Gateway Technology (Thermo Fisher Scientific). In brief, the fragment of interest was amplified by PCR to introduce attB sites. This was cloned into a pDONR221 vector using the BP clonase II (11789020, Thermo Fisher Scientific). The fragment of interest was further subcloned into a pHygro vector containing a hygromycin B-resistance cassette, respectively (gift of J. Silva, Stem Cell Institute) for expression in mammalian cells. The recombination reaction was carried out using the LR Clonase II (11791100, Thermo Fisher Scientific). Plasmid generated was then transformed into DH5α cells using the heat shock method and amplified. Sequencing was performed to confirm the sequence of the plasmid DNA. Stable transfection of mESC was carried out using Lipofectamine 3000 transfection reagent kit (L3000001, Thermo Fisher Scientific). Here, the day before transfection 50,000 cells were plated on 24-well gelatin-coated plates in N2B27 2iLIF medium without antibiotics. For transfection, 0.5 μg of a PiggyBac transposon vector (gift of J. Silva, Stem Cell Institute) and 0.5 μg of Ecad-GFP was used. Transfected cells were selected with 10 μg/ml hygromycin and the resulting colonies were manually picked and expanded.

Propagation of mouse embryonic stem cells

mESCs were propagated in FC medium supplemented with 2iLIF at 37°C, 5% CO_2, 21% O_2. Medium was changed every other day. Cells were passaged every 2-3 days depending on the confluency: colonies were washed in PBS, then treated with 0.05% trypsin-EDTA (25300054, Thermo Fisher Scientific) for 3-4 min at 37°C, the enzymatic reaction was stopped by FC medium addition containing serum to inactivate the enzyme and finally centrifugated at 1,000 rpm for 5 min. Pellet was then resuspended in single cells in fresh FC medium supplemented with 2iLIF and plated gelatin-coated plates (150628, Thermo Fisher Scientific). Cells were routinely tested for mycoplasma contamination by PCR.

3D culture of mouse embryonic stem cells

For all the experiments presented, single mESCs were embedded in 3D Matrigel (356230, BD Biosciences) and cultured in N2B27 with or without 2iLIF, to induce exit or preserve naive pluripotency respectively, in 8-well μ-Slides (80826, Ibidi). For this, cells were dissociated by trypsin-EDTA. The cell pellet obtained after the stop of the trypsinization reaction was resuspended in PBS to measure the cell concentration. Then, the suspension containing 20,000 cells was centrifuged, the resulting pellet resuspended in 20 μL of ice-cold Matrigel and placed as a drop in a well of an ibidi dish and incubated for 5 min at 37°C to allow the Matrigel to solidify. Next, 300 μL of medium (N2B27) were added to the well. 2iLIF was supplemented in N2B27 to induce preservation naive pluripotency. For the rescue experiment (Figure 5): ROCK inhibitor (Y27632, Stem Cell Technology, 72304, resuspended in DMSO) was supplemented in N2B27 at 20 μM for initial 48h and 40 μM for the final 24h; GSK3i at 0.75 μM (Chiron, CHIR99021, Stem Cell Institute Cambridge); FGF2 at 50 ng/ml (Stem Cell Institute Cambridge) and IGF1 at 100 ng/ml (Stem Cell 78078, resuspended in water).

Fixation

Embryos and mESCs were fixed in 4% paraformaldehyde (11586711, Electron Microscopy Sciences) for 20 min and washed in PBS containing 0.1% Tween (P1379, Sigma). Methanol fixation was performed by immersing cells in −20°C cold DMSO:MeOH (1:5), incubated for 30 min at 4°C.

Immunofluorescence staining

Following fixation, embryos and mESCs were permeabilized for 30 min in PBS containing 0.3% Triton X-100 and 0.1 M glycine for 30 min at room temperature. No blocking step was performed. Samples were then incubated overnight at 4°C in primary antibodies diluted in PBS containing: 10% FBS, 2% bovine serum albumin (BSA), and 0.1% Tween-20. A volume of primary antibody solution between 100-150 μL was used per sample. Samples were then washed in PBS (0.1% Tween-20) and incubated for 2h at room temperature protected from light in fluorescently conjugated Alexa Fluor secondary antibodies 1:500 (ThermoFisher Scientific) and DAPI (D3571, ThermoFisher Scientific, dilution 1/500), diluted in PBS (10% FBS, 2% BSA, 0.1% Tween-20). Samples were stored at 4°C in PBS supplemented with 0.1% sodium azide to prevent fungal or bacterial growth.

Primary antibodies used. We have not characterized or performed validation of antibody but have chosen antibodies where target antigen, cross reactivity, validation by ELISA and Western Blot have been tested and provided by the manufacturer. Details for each antibody is also provided in the Key resources table. Primary antibodies: Integrin β1 for mouse embryos and mESCs (rat, 1:150, MAB1997). Integrin β1 for human embryos (rat, 1:50, MABT821). pMLC-II (rabbit, 1:100, Cell Signaling 3671S, 519, Ser19). Par3 (rabbit, 1:100, MERCK Millipore 07-330). Par6 (rabbit, 1:200, Santa Cruz SC-67393). Ecad (rat, 1:200, Thermo Fisher Scientific 13-1900). Podocalyxin (rat, 1:500, R&D Systems, MAB1556). Golgi (GM130) (mouse, 1:200, BD Biosciences BD610822). γ-Tubulin (mouse, 1:500, Sigma T6557), only on methanol fixed samples. Cleaved-Caspase 3 (rabbit, 1:200, Cell Signaling 9664, D175, 5A1E). Otx2 (goat, 1:250, R&D Systems AF1979). GFP to detect Ecad-GFP (rat, 1:1000, GF090R). PKC (mouse, 1:50 Santa Cruz SC-17781). Laminin (Rabbit, 1:200, Sigma-Merck L9393). Perlecan (Rat, 1:100, Merck - MAB1948P). Oct4 (mouse monoclonal, 1:200, Santa Cruz, sc5279). Nanog (rabbit, 1:200, Abcam, ab80892). Phospho-Histone H3 (pHH3) (rabbit polyclonal, 1:500, Merck, 06-570)

Secondary antibodies used (1:500, ThermoFisher Scientific): Alexa Fluor 488 Donkey anti-Rat, A-21208. Alexa Fluor 594 Donkey anti-Rat, A21209. Alexa Fluor 647 Donkey anti-Rat, A21247. Alexa Fluor 488 Donkey anti-Goat, A-11055. Alexa Fluor 568 Donkey anti-Goat, A-11057. Alexa Fluor 647 Donkey anti-Goat, A21447. Alexa Fluor 488 Donkey anti-Mouse, A21202. Alexa Fluor 568 Donkey anti-Mouse, A10037. Alexa Fluor 647 Donkey anti-Mouse, A31571. Alexa Fluor 488 Donkey anti-Rabbit, A21206. Alexa Fluor 568 Donkey anti-Rabbit, A10042. Alexa Fluor 647 Donkey anti-Rabbit, A31573.

Phalloidins: Alexa Fluor 488 Phalloidin (1:500, Thermo Fisher Scientific, A12379); Alexa Fluor 594 Phalloidin (1:500, Thermo Fisher Scientific, A12381), Alexa Fluor 647 Phalloidin (1:250, Thermo Fisher Scientific, A22278).

Time-lapse movies

Ecad-GFP cells (mESCs integrin β1 (*fl/fl*) and (*Δ/Δ*)) were seeded in matrigel under differentiating conditions in N2B27 without 2iLIF. 24-hours post seeding, cells were imaged at a time frame of 10 min per frame and z-resolution of 1 μm on Leica SP8 confocal microscope using 63x oil objective.

QUANTIFICATION AND STATISTICAL ANALYSIS

Image acquisition

Fixed mESCs and embryos were imaged on a Leica SP8 confocal microscope using 25x water, 40x oil and 63x oil objectives. Images were acquired with a z-step size of 0.6 μm and a 2x line average.

Quantifications

Post-acquisition processing of raw files was carried out using the Fiji software (Schindelin et al., 2012) for brightness adjustments, cropping, outlier removal and quantifications. Fluorescence intensity quantification as presented in Figures 3E, 5E, and 5G was performed by measuring the mean gray values of pMLC-II on the middle plane along the Z axis of the epiblast/ESC spheroid by Fiji. Mean gray values were measured by a segmented line (width 20, spline fit) or ellipse selection at two sites: along the basal perimeter and

along the apical domain of the epiblast. Values reported are ratios of the apical versus basal values of pMLC-II. Values above 1 indicates enrichment of pMLC-II at the apical site; values below 1 indicates enrichment of pMLC-II basally. Polarization (polarized versus un-polarized) was determined on the relative localization of Golgi (GM130) and PAR3. The nuclear-centrosome angle was quantified relative to the midline axis between two cells. Quantification of apoptosis refers to the presence of c-caspase+ cells within individual mESC structures or epiblasts: the percentage displayed thus refers to the number of mESC or epiblasts positive for c-caspase. The number of c-caspase+ cells, normalized for the total epiblast cell number, is presented instead in Figure 1I and refers to the percentage of c-caspase+ cells within each epiblast analyzed. Mitotic index is calculated as the number of phospho-histone H3 positive cells divided the total cell number of cells in each spheroid. Quantification of the presence of cavity and lumen in embryos and mESCs is based on Phalloidin staining. Quantification in Figure 1G refers to the assessment of the presence or absence of the epiblast lineage (OCT4) in embryos recovered at E5.5. For all quantifications a minimum of three independent experiments was performed. The exact n number is provided in the figure legend of each experiment.

Statistical analysis

Statistical details for each experiment can be found in the figure legends. Statistical data analysis was performed using GraphPad Prism software (version 7). Comparison between two groups was performed through the non-parametric Mann-Whitney Test. Normality was tested by D'Agostino-Pearson omnibus test. Comparison between frequencies was tested by Fisher's Exact test. Statistical significance was defined as follows: $p \leq 0.05$ was considered statistically significant (*), $p \leq 0.01$ (**), $p \leq 0.001$ (***), $p \leq 0.0001$ (****). Sample size (n) was defined as number of embryos or mESC structures used in each analysis. Definition of center and dispersion: the mean ± SEM (standard error of the mean) is presented in all bar and dot plots. In box and whisker plot: the box represents the 25th and 75th percentiles interval, the line in the middle of the box the median, the cross represents the mean, whiskers show the minimum and maximum values. Statistical details such as p value, n number, and statistical test of every experiment can be found in the corresponding figure legend. No statistical method was used to predetermine sample size. The investigators were not blinded to group allocation during experiments and outcome assessment.

Adobe Illustrator was used for panel assembly and schematics.

Cell Reports, Volume *34*

Supplemental information

Integrin β1 coordinates survival and morphogenesis of the embryonic lineage upon implantation and pluripotency transition

Matteo Amitaba Molè, Antonia Weberling, Reinhard Fässler, Alison Campbell, Simon Fishel, and Magdalena Zernicka-Goetz

SUPPLEMENTAL INFORMATION:

Integrin β1 coordinates survival and morphogenesis of the embryonic lineage upon implantation and pluripotency transition

Matteo Amitaba Molè[1†], Antonia Weberling[1†], Reinhard Fässler[2], Alison Campbell[3], Simon Fishel[3,4], Magdalena Zernicka-Goetz[1,5,6,*]

Figure S1

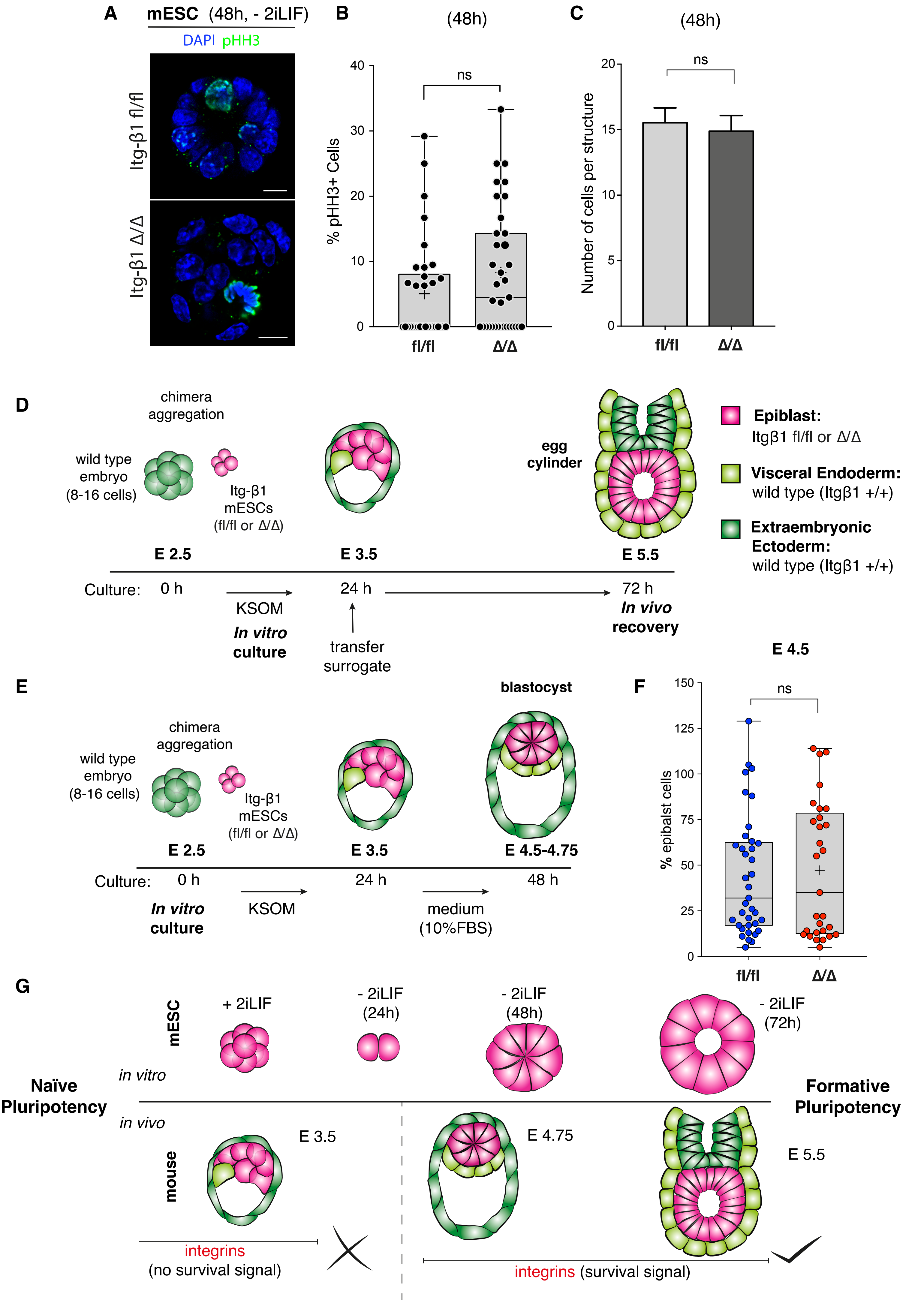
A
mESC (48h, - 2iLIF)
DAPI pHH3
Itg-β1 fl/fl
Itg-β1 Δ/Δ
B
(48h)
% pHH3+ Cells
ns
fl/fl
Δ/Δ
C
(48h)
Number of cells per structure
ns
fl/fl
Δ/Δ
D
chimera aggregation
wild type embryo (8-16 cells)
Itg-β1 mESCs (fl/fl or Δ/Δ)
egg cylinder
Epiblast: Itgβ1 fl/fl or Δ/Δ
Visceral Endoderm: wild type (Itgβ1 +/+)
Extraembryonic Ectoderm: wild type (Itgβ1 +/+)
E 2.5
E 3.5
E 5.5
Culture: 0 h
KSOM
In vitro culture
24 h
transfer surrogate
72 h
In vivo recovery
E
chimera aggregation
wild type embryo (8-16 cells)
Itg-β1 mESCs (fl/fl or Δ/Δ)
blastocyst
E 2.5
E 3.5
E 4.5-4.75
Culture: 0 h
In vitro culture
KSOM
24 h
medium (10%FBS)
48 h
F
E 4.5
% epibalst cells
ns
fl/fl
Δ/Δ
G
mESC
+ 2iLIF
- 2iLIF (24h)
- 2iLIF (48h)
- 2iLIF (72h)
in vitro
Naïve Pluripotency
Formative Pluripotency
in vivo
mouse
E 3.5
E 4.75
E 5.5
integrins (no survival signal)
integrins (survival signal)

Figure S1. Proliferation and methods for the generation of chimeric embryos. Related to Figure 1. (**A**), Cells undergoing mitosis as revealed by phospho-histone H3 staining in wild type (fl/fl, upper) and mutant (Δ/Δ, lower) mESC spheroids at 48-hours culture. Scale bars: 10 μm. (**B**), Quantification of mitotic index as the percentage of phospho-histone H3 positive cells of the total cell number in each spheroid. Mann-Whitney Test, p=ns, number of spheroids n=34 (fl/fl), n=35 (Δ/Δ); number of replicates=3. (**C**), Comparison of the total number of cells in each spheroid. Mann-Whitney Test, p=ns. (**D**), Embryos recovered at E2.5 (8-16 cells stage) from wild type embryos are aggregated with mESCs either wild type (fl/fl) or mutant (Δ/Δ) for *Itgβ1* locus. Embryos are cultured for 24-hours in KSOM and then transferred into E2.5 pseudo-pregnant surrogate mice. After 72-hours embryos are recovered by dissection. Extraembryonic lineages (ExE and VE) are derived from wild type embryos, while the epiblast compartment from mESCs homozygous for either the floxed (fl/fl) or deleted allele (Δ/Δ) of *Itgβ1*. (**E**), Culture method to generate chimeric embryos at the expanded blastocyst stage E4.5-4.75: embryos recovered at E2.5 (8-16 cells stage) from wild type embryos are aggregated with mESC either wild type (fl/fl) or mutant (Δ/Δ), cultured for 24-hours in KSOM and transferred into IVC (*in vitro* culture) medium supplemented with 10% FBS for further 24-hours. (**F**), Comparison of the total number of epiblast cells in chimeric blastocysts at E4.5-4.75 between wild types (Epi:fl/fl) and mutants (Epi:Δ/Δ) as refereed Figure 1 H-J: Mann-Whitney Test, p=ns (number of embryos n=37 (fl/fl), n=29 (Δ/Δ)). (**G**), Schematics of integrin requirement throughout development in mESCs (upper) and mouse embryos (lower). Integrin signalling is dispensable during early pre-implantation development corresponding to the *in vitro* state of naïve pluripotency. Conversely, signaling from integrin-mediated adhesion ensures survival of the epiblast upon implantation and transition towards the formative state of pluripotency. Scale bar: 10 μm (A).

Figure S2

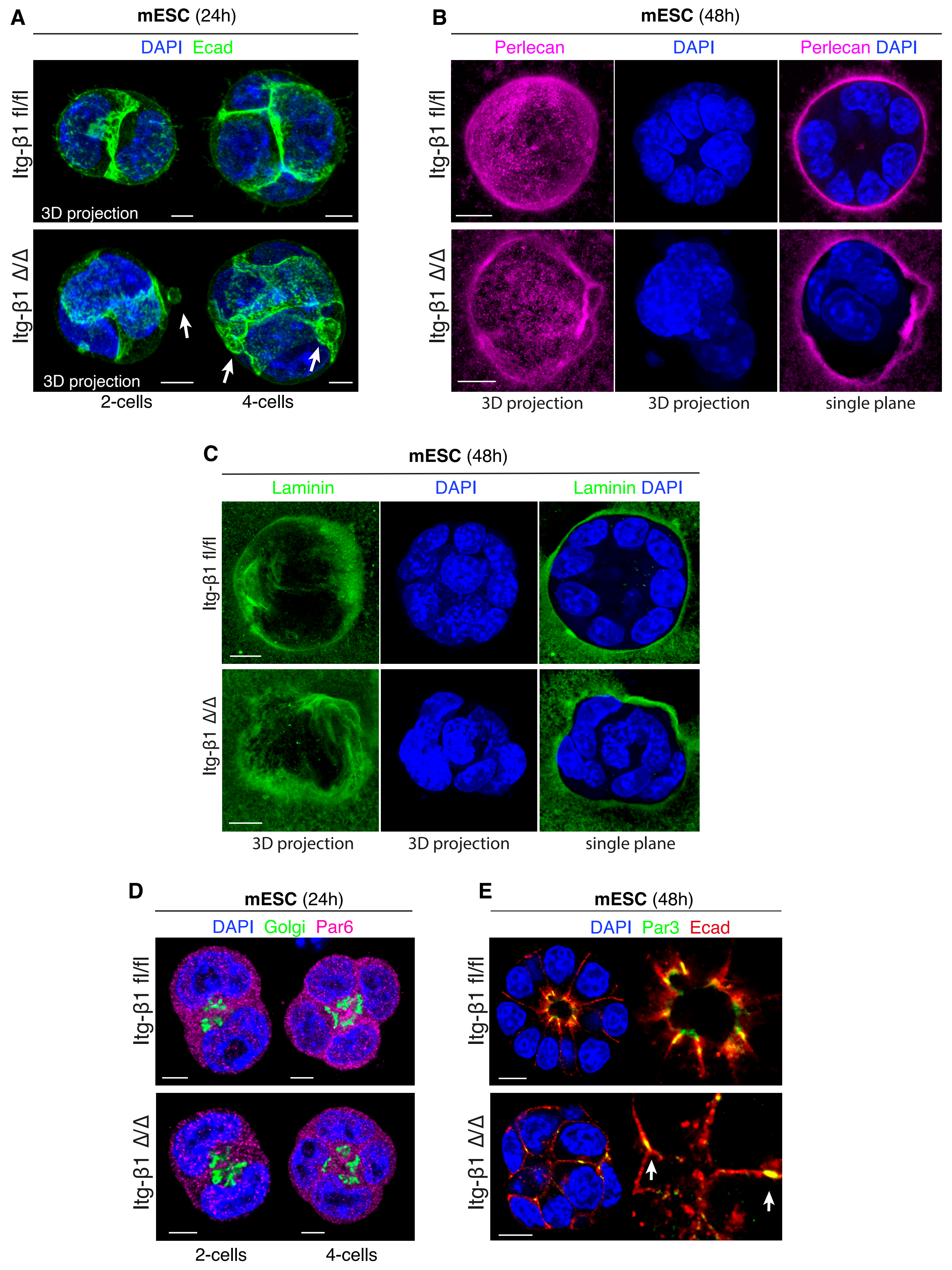
A
mESC (24h)
DAPI Ecad
Itg-β1 fl/fl
Itg-β1 Δ/Δ
3D projection
3D projection
2-cells
4-cells
B
mESC (48h)
Perlecan
DAPI
Perlecan DAPI
Itg-β1 fl/fl
Itg-β1 Δ/Δ
3D projection
3D projection
single plane
C
mESC (48h)
Laminin
DAPI
Laminin DAPI
Itg-β1 fl/fl
Itg-β1 Δ/Δ
3D projection
3D projection
single plane
D
mESC (24h)
DAPI Golgi Par6
Itg-β1 fl/fl
Itg-β1 Δ/Δ
2-cells
4-cells
E
mESC (48h)
DAPI Par3 Ecad
Itg-β1 fl/fl
Itg-β1 Δ/Δ

Figure S2. Loss of integrin β1 causes basal blebs but is dispensable for apico-basal polarity establishment or basement membrane assembly. Related to Figure 3. (**A**), Blebs are observed after 24-hours culture on the basal domain of mutant mESCs (arrows). (**B**-**C**), Immunofluorescences staining for the basement membrane (BM) components Perlecan (B) and pan-Laminins (C) at 48-hours culture. Mutant mESCs (Itgβ1 Δ/Δ) assemble correctly the BM on the basal side, similarly to wild types (Itgβ1 fl/fl). (**D**), Both wild type and mutant cells orient the Golgi apparatus towards the apical site. PAR6 complex is not expressed at 24-hours. (**E**) Assessment of polarity at 48-hours by the localization of PAR3. In wild type cells, PAR3 is recruited at apical junctions at the site of E-cadherin expression. In mutant cells, PAR3 maintains a junctional localization, as opposed to basal PAR6 (see Figure 4H). Scale bars: 5 μm (A, D), 10 μm (B-C, E).

Figure S3

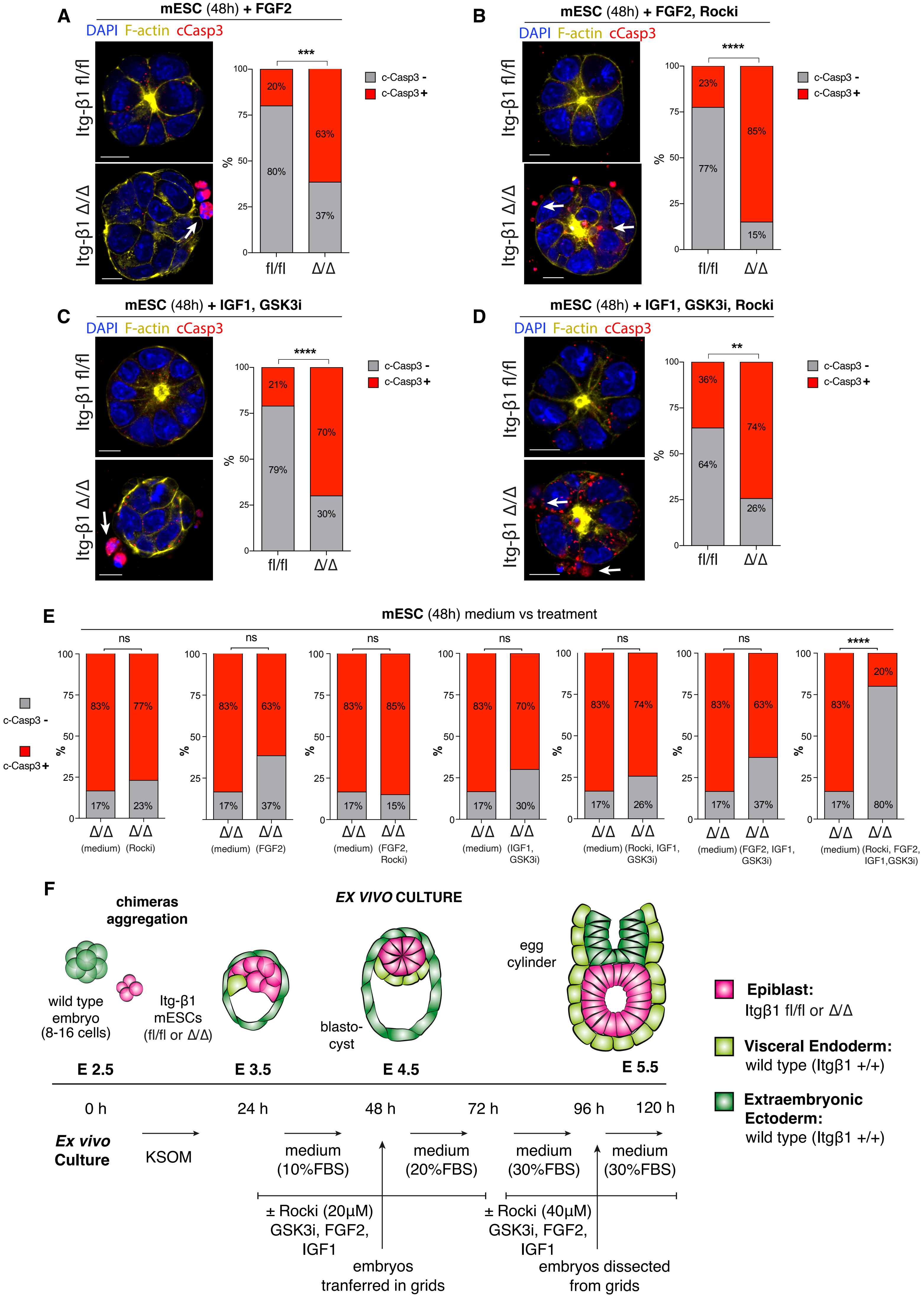
A
mESC (48h) + FGF2
DAPI F-actin cCasp3
Itg-β1 fl/fl
Itg-β1 Δ/Δ
***
20%
80%
63%
37%
%
fl/fl
Δ/Δ
c-Casp3 -
c-Casp3 +
B
mESC (48h) + FGF2, Rocki
DAPI F-actin cCasp3
Itg-β1 fl/fl
Itg-β1 Δ/Δ
****
23%
77%
85%
15%
%
fl/fl
Δ/Δ
c-Casp3 -
c-Casp3 +
C
mESC (48h) + IGF1, GSK3i
DAPI F-actin cCasp3
Itg-β1 fl/fl
Itg-β1 Δ/Δ
****
21%
79%
70%
30%
%
fl/fl
Δ/Δ
c-Casp3 -
c-Casp3 +
D
mESC (48h) + IGF1, GSK3i, Rocki
DAPI F-actin cCasp3
Itg-β1 fl/fl
Itg-β1 Δ/Δ
**
36%
64%
74%
26%
%
fl/fl
Δ/Δ
c-Casp3 -
c-Casp3 +
E
mESC (48h) medium vs treatment
c-Casp3 -
c-Casp3 +
ns
83% 77%
17% 23%
Δ/Δ (medium) Δ/Δ (Rocki)
ns
83% 63%
17% 37%
Δ/Δ (medium) Δ/Δ (FGF2)
ns
83% 85%
17% 15%
Δ/Δ (medium) Δ/Δ (FGF2, Rocki)
ns
83% 70%
17% 30%
Δ/Δ (medium) Δ/Δ (IGF1, GSK3i)
ns
83% 74%
17% 26%
Δ/Δ (medium) Δ/Δ (Rocki, IGF1, GSK3i)
ns
83% 63%
17% 37%
Δ/Δ (medium) Δ/Δ (FGF2, IGF1, GSK3i)
****
83% 20%
17% 80%
Δ/Δ (medium) Δ/Δ (Rocki, FGF2, IGF1,GSK3i)
%
F
chimeras aggregation
EX VIVO CULTURE
wild type embryo (8-16 cells)
Itg-β1 mESCs (fl/fl or Δ/Δ)
blasto-cyst
egg cylinder
E 2.5
E 3.5
E 4.5
E 5.5
0 h
24 h
48 h
72 h
96 h
120 h
Ex vivo Culture
KSOM
medium (10%FBS)
medium (20%FBS)
medium (30%FBS)
medium (30%FBS)
± Rocki (20µM) GSK3i, FGF2, IGF1
± Rocki (40µM) GSK3i, FGF2, IGF1
embryos tranferred in grids
embryos dissected from grids
Epiblast: Itgβ1 fl/fl or Δ/Δ
Visceral Endoderm: wild type (Itgβ1 +/+)
Extraembryonic Ectoderm: wild type (Itgβ1 +/+)

Figure S3. Pharmacological treatments, impact on morphogenesis and survival. Related to Figure 5 and 6. (**A**), Supplementation of FGF2 alone does not prevent initiation of apoptosis in integrin β1 mutant cells (Δ/Δ): Fisher's Exact Test: *** p=0.0004 (number of spheroids n=35 (fl/fl), n=39 (Δ/Δ), 3 replicate experiments). (**B**), Supplementation of FGF2 + Rock inhibitor does not prevent initiation of apoptosis in integrin β1 mutant cells (Δ/Δ): Fisher's Exact Test: **** p<0.0001 (number of spheroids n=40 (fl/fl), n=40 (Δ/Δ), 3 replicate experiments). (**C**), Supplementation of IGF1 + GSK3 inhibitor does not prevent initiation of apoptosis in integrin β1 mutant cells (Δ/Δ): Fisher's Exact Test: **** p<0.0001 (number of spheroids n=38 (fl/fl), n=40 (Δ/Δ), 3 replicate experiments). (**D**), Supplementation of IGF1 + GSK3 inhibitor + Rock inhibitor does not prevent initiation of apoptosis in integrin β1 mutant cells (Δ/Δ): Fisher's Exact Test: ** p=0.0013 (number of spheroids n=39 (fl/fl), n=39 (Δ/Δ), 3 replicate experiments). (**E**) Comparison of the Δ/Δ mESCs in medium without supplementation vs. all conditions tested at 48-hours: from left to right Rocki alone, FGF2 alone, FGF2+Rocki, IGF1+GSK3i, Rocki+IGF1+GSK3i, FGF2+ IGF1+GSK3i, and complete supplementation with Rocki+FGF2+ IGF1+GSK3i. Treatment with each combination is not able to rescue the survival and does not differ from Δ/Δ mESCs in medium only (Fisher's Exact Test: p=ns). Only the supplementation of the four factors together (Rocki+FGF2+ IGF1+GSK3i) is able to rescue the survival of the mutant mESCs (Δ/Δ in medium vs Δ/Δ in full supplementation: Fisher's Exact Test: ****p<0.0001), to a comparable level to wild type (Figure 5D, Fisher's Exact Test: p=ns). (**F**), Method of culture from pre- to post implantation: blastocysts are recovered at E2.5 (8-16 cells stage) from wild type embryos and aggregated with mESC wild type (fl/fl) or mutant (Δ/Δ) for *Itgβ1* locus. Embryos are cultured for 24-hours in KSOM in drops. Expanded blastocysts are moved in grid mesh in medium with increasing concentration of FBS supplemented as shown. FGF2, IGF1 and GSK3i are supplemented in medium after 24-hours. ROCKi is supplemented in medium after 24-hours at 20 μM and increased to 40 μm during the last 48-hours culture. See methods for more details. Scale bars: 10 μm (A-E)
